# Customized Hydrogel System for the Spatiotemporal Sequential Treatment of Periodontitis Propelled by ZEB1

**DOI:** 10.1002/advs.202503338

**Published:** 2025-04-04

**Authors:** Jiafei Chen, Xiaoxu Guan, Lina Chen, Bingzhu Zheng, Feiyu Li, Chao Fang, Yike Fu, Xiang Li, Huiming Wang, Yi Zhou

**Affiliations:** ^1^ The Affiliated Hospital of Stomatology School of Stomatology Zhejiang University of Medicine and Key Laboratory of Oral Biomedical Research of Zhejiang Province Hangzhou 310006 China; ^2^ State Key Laboratory of Silicon and Advanced Semiconductor Materials School of Materials Science and Engineering Zhejiang University Hangzhou 310058 China; ^3^ Department of Cardiology Shaoxing Central Hospital Shaoxing Zhejiang Province 312000 China; ^4^ ZJU‐Hangzhou Global Science and Technology Innovation Center Zhejiang University Hangzhou 311215 China; ^5^ iBioMat PharmTeck (Hangzhou) Co. Ltd. Building C 3F, 2959 Yuhangtang Road Hangzhou 311100 China; ^6^ Department of Medical Oncology The First Affiliated Hospital Zhejiang University School of Medicine Hangzhou 310003 China

**Keywords:** antibacterial, anti‐inflammatory, bone regeneration, immune regulation, ZEB1

## Abstract

Advanced periodontitis initiates with *Porphyromonas gingivalis* (*P. gingivalis*) infection, which subsequently triggers chronic inflammation, immune imbalance, and ultimately causes alveolar bone resorption. Traditional periodontal treatment focuses on the elimination of triggering factors, but tend to ignore the improvement of the inflammatory microenvironment and the remodeling of the osteogenic mineralization space. Herein, zinc‐aluminum layered double hydroxide nanosheets (LDHs) loaded with icariin (ICA) are encapsulated into a gallic acid (GA)‐modified hydroxybutyl chitosan hydrogel (GA‐HBC), giving rise to a customized hydrogel system named GA‐HBC‐LIC, which can sequentially actualize antibacterial, anti‐inflammatory, and remineralization functions. A neutral chemical‐humoral space is created for osteogenesis via means of sequential regulation by the smart hydrogel. Concomitantly, appropriate mechanical properties and degradation performance of the hydrogel provide a desirable physical space for remineralization. In the spatiotemporal modulation of the hydrogel, zinc finger E‐box‐binding homeobox 1 (ZEB1) target of released zinc ions (Zn^2+^) action promotes macrophage polarization from M1 to M2 phenotype, thereby remodeling the immune microenvironment and releasing cytokines conducive to tissue regeneration. In sum, this study highlights the critical role of sequential inflammation regulation and the maintenance of osteogenic space in the regeneration of periodontal tissues, offering new insights for the clinical management of periodontitis.

## Graphical abstract

Through elaborate design, a biodegradable scaffold with excellent mechanical properties and suitability is fabricated, providing spatial support for subsequent physiological activities. The prepared GA‐HBC‐LIC hydrogel possesses sequential antibacterial, antioxidant, anti‐inflammatory and immunomodulatory functions, offering a suitable chemical and humoral microenvironment for effective bone regeneration. Customized hydrogel system represented a milestone in the promotion of healing in inflammatory and infectious bone defects in a spatiotemporal manner.

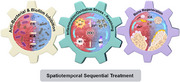

## 1 Introduction

Periodontitis, the primary etiology of dental loss, exhibits significant associations with various systemic illnesses and presents a formidable obstacle to human well‐being.^[^ ^1^ ^]^ The development of periodontitis entails a sequence of advancing pathological mechanisms, incorporating infection, inflammation, oxidative stress, immune dysregulation, and impaired functions of bone marrow mesenchymal stem cells.^[^ ^2^ ^]^ Currently, the common clinical therapies are scaling and root planning (SRP) and antibiotic treatment, which narrowly focus on eliminating the pathogens while overlooking the importance of reestablishing periodontal homeostasis.^[^ ^3^ ^]^ Thus, there is an urgent need to develop an comprehensive approach for holistic intervention. Considering the episodic progression of periodontitis, a sequential therapeutic is innovatively proposed here, which divides the treatment of periodontitis into three interrelated stages: antimicrobial treatment, inflammation modulation, and periodontal restoration. Initially, the infection must be thwarted by eradicating pathogenic bacteria in the affected region. Subsequently, equilibrium in oxidative stress must be restored and the immune microenvironment must be modulated to achieve an anti‐inflammatory state.^[^ ^4^ ^]^ Eventually, an ideal chemical‐humoral environment should be created to promote the regeneration of periodontal bone tissue, thereby realizing the ideal multi‐faceted and synergistic treatment.^[^ ^5^ ^]^ Meanwhile, due to the formation of periodontal pockets, to further enhance bone repair, it is necessary to provide physical space support, and local drug delivery system (LDDS) serves as an ideal choice.^[^ ^6^ ^]^

Hydroxybutyl chitosan hydrogel (HBC) is an injectable thermosensitive hydrogel with exceptional biocompatibility, and widely used in LDDS.^[^ ^7^ ^]^ It is evident that HBC hydrogel holds significant promise in the management of periodontitis.^[^ ^8^ ^]^ GA exhibits broad‐spectrum antibacterial properties and anti‐inflammatory activities.^[^ ^9^ ^]^ Using GA to modify HBC can further augment the aforementioned functionalities of the hydrogel system and inhibit the burst release of GA in vivo.^[^ ^10^ ^]^ GA‐HBC hydrogel also amalgamates appropriate mechanical properties and degradation properties, allowing it to remain in the periodontal pocket space for an extended duration. However, the acidic degradation products of GA‐HBC hydrogel may hinder subsequent inflammation control and tissue regeneration.^[^ ^11^ ^]^ ZnAl‐LDHs are weakly alkaline nanosheets that can slowly release Zn^2^⁺ within the GA‐HBC matrix.^[^ ^12^ ^]^ Zn^2^⁺ plays a crucial role in regulating macrophage function. Studies have elucidated its ability to prompt macrophage polarization toward the M2 phenotype via the phosphatidylinositol 3‐kinase/protein kinase B (PI3K/Akt) pathway.^[^ ^13^ ^]^ To further enhance osteogenic activity, ICA, which promotes osteogenesis while inhibiting bone resorption, was loaded onto LDHs.^[^ ^14^ ^]^ Through electrostatic adsorption, its burst release was prevented, enabling a sustained stimulation of new bone regeneration.^[^ ^15^ ^]^

**Figure 1:**
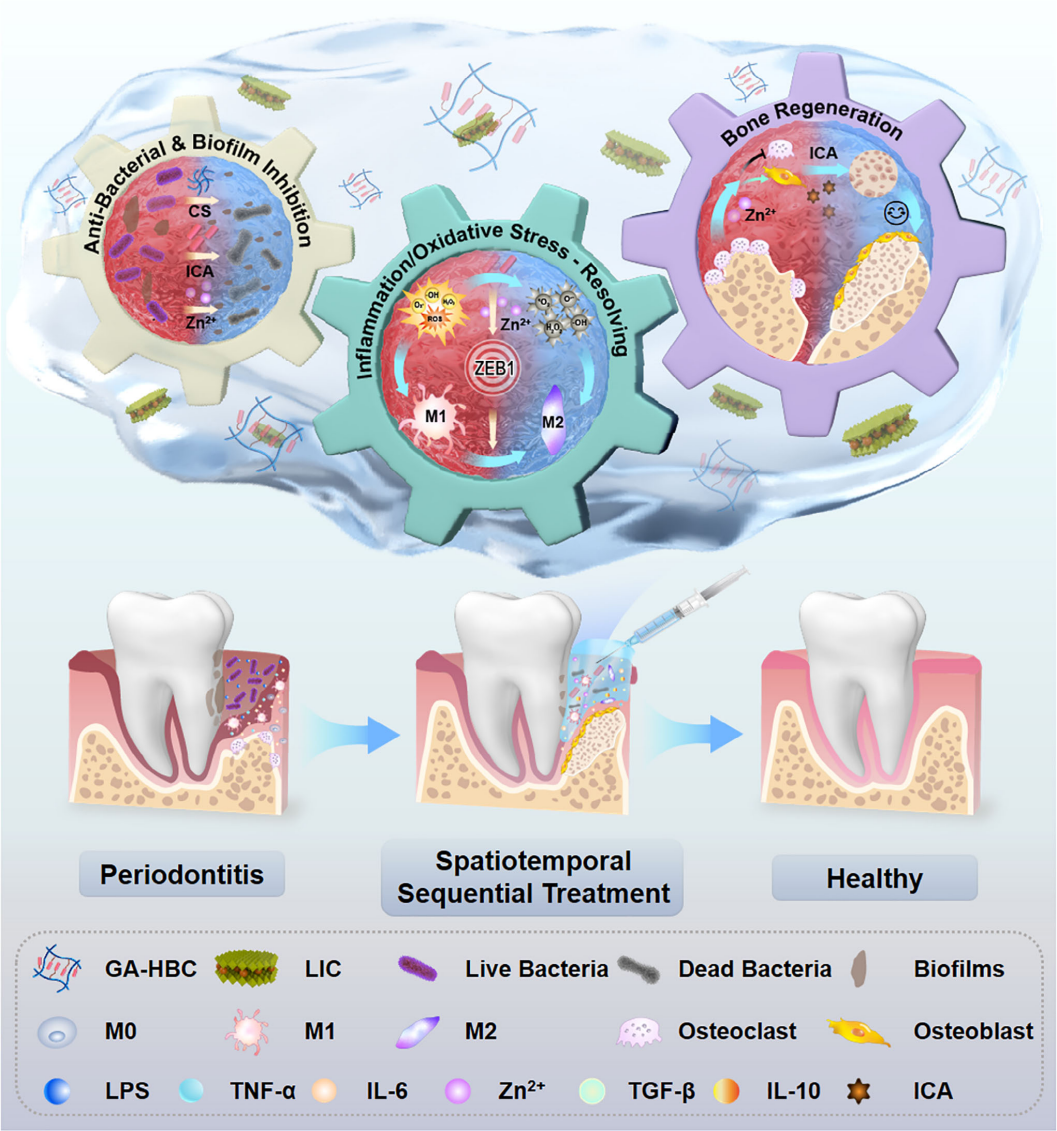
Schematic diagram of the customized GA‐HBC‐LIC hydrogel promoting the healing of chronic periodontitis through spatiotemporal dynamic sequential therapy.

In this research, we customized a treatment system based on the progression of periodontitis to achieve spatiotemporal regulation. First, the GA‐HBC‐LIC hydrogel eliminated bacteria through the multi‐pathway synergistic effects of chitosan (CS), GA, and Zn^2^⁺. Subsequently, the intelligent and gradual release of Zn^2^⁺ targeted ZEB1, transforming macrophages from the M1 to M2 phenotype and establishing an anti‐inflammatory microenvironment. Finally, ICA promoted bone regeneration by locally and continuously releasing the compound and effectively collaborating with Zn^2^⁺. The findings demonstrated that GA‐HBC‐LIC reshaped the chemical‐humoral landscape of periodontitis through sequential regulation and provided physical support, efficiently enhancing tissue restoration. In conclusion, this approach achieved dynamic spatiotemporal treatment of periodontitis by leveraging the synergistic properties of its functional constituents (**Figure** **1**).

## 2 Results and Discussion

### 2.1 Preparation and Characterization of the Hydrogel System

**Figure 2:**
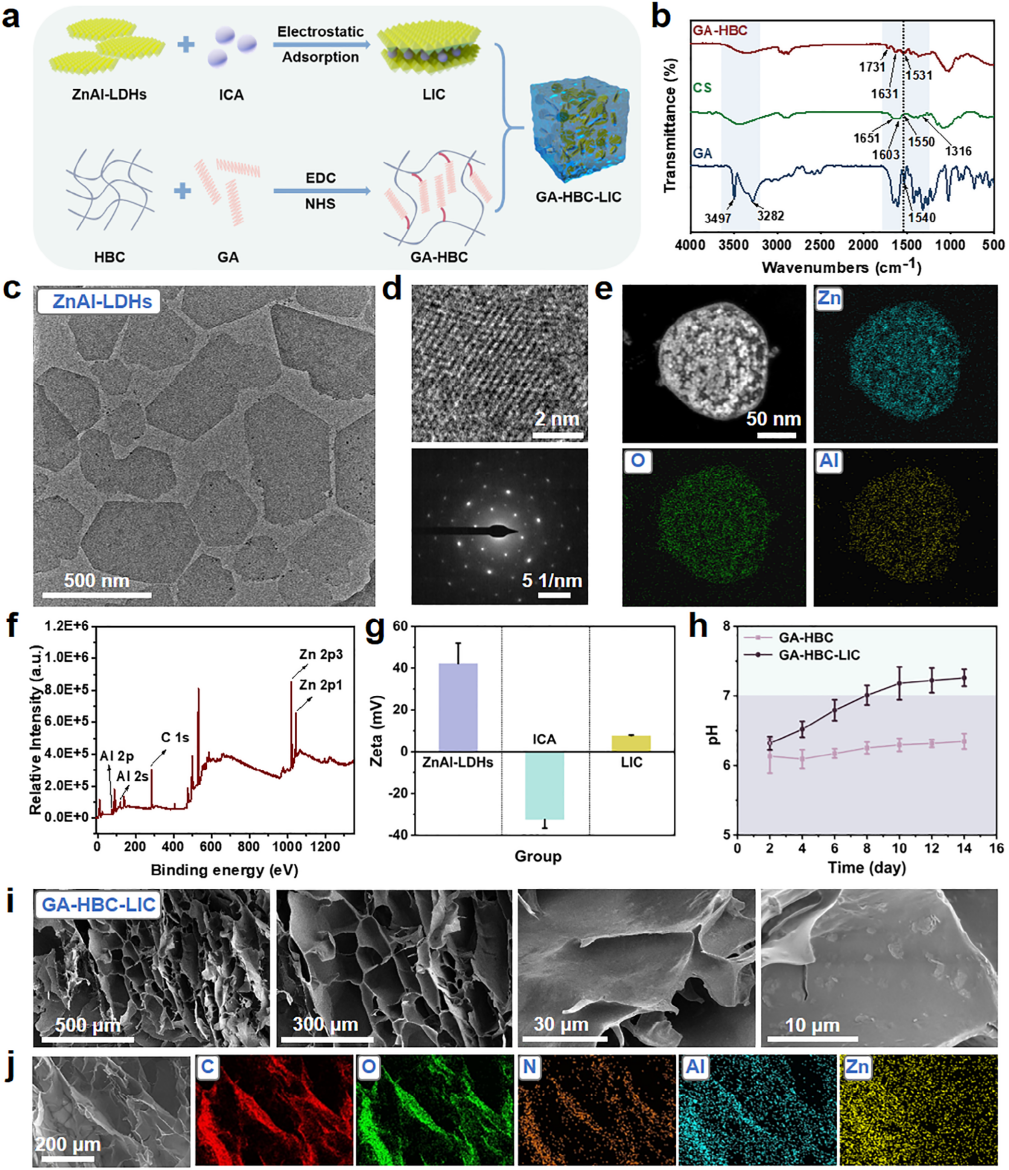
Preparation and characterization of the hydrogel system. a) Schematic illustration of the preparation process for GA‐HBC‐LIC hydrogel. b) FT‐IR spectra of GA, CS and GA‐HBC. c) TEM image of ZnAl‐LDHs. d) HR‐TEM image and SAED pattern of ZnAl‐LDHs. e) EDS‐mapping images of ZnAl‐LDHs. f) XPS full spectrum of ZnAl‐LDHs. g) Zeta potential of ZnAl‐LDHs, ICA, and LIC. h) Temporal variation of pH values in the supernatants of GA‐HBC and GA‐HBC‐LIC hydrogels. i) SEM micrographs of the GA‐HBC‐LIC hydrogel. j) Element mapping of the GA‐HBC‐LIC hydrogel.

GA‐HBC‐LIC was obtained by encapsulating ICA‐loaded LDHs (LIC) into the GA‐modified HBC hydrogel (**Figure** **2**a). Fourier transform infrared spectroscopy (FT‐IR) spectroscopy analysis demonstrated that GA was conjugated at the amino and hydroxyl groups of CS, verifying the successful preparation of GA‐HBC hydrogel (Figure 2b; Figure S1a, Supporting Information).^[^ ^16^ ^]^ Transmission electron microscopy (TEM) showed that the LDHs was ≈300–500 nm in size and presented hexagonal sheet structure (Figure 2c). High‐resolution TEM (HR‐TEM) images and the selected area electron diffraction (SAED) pattern exhibited that the LDHs exhibited well‐defined lattice fringes, confirming their single‐crystal structure (Figure 2d). Energy‐dispersive X‐ray (EDX) elemental mapping revealed uniform distribution of Zn and Al elements within the nanosheets, with a Zn/Al molar ratio of 1.76 (Figure 2e, Table S1, Supporting Information). The hydrodynamic diameters of LDHs were determined by dynamic light scattering (DLS) to be ≈401.3 nm in water and 653.8 nm in dulbecco's modified eagle medium (DMEM), respectively (Figure S1b, Supporting Information). X‐ray photoelectron spectroscopy (XPS) analysis revealed distinct elemental signals for Al^3+^ and Zn^2+^ on the surface of LDHs (Figure 2f; Figure S1c,d, Supporting Information). Conclusively, all the results evinced the successful fabrication of LDHs. Research has shown that the direct incorporation of ICA into hydrogel matrices leads to rapid release within a short timeframe, typically within ≈72 h.^[^ ^17^ ^]^ Additionally, the inherent hydrophobic nature of ICA may constrain the uniformity and quantity of its loading in hydrophilic gels.^[^ ^18^ ^]^ To extend the retention duration of ICA and enhance sustained impetus for new bone regeneration, negatively charged ICA was combined with LDHs via electrostatic interactions. The zeta potential of LIC was measured, and the loading efficiency of ICA was established at 84.82% (Figure 2g; Figure S1e,f, Supporting Information). Furthermore, through the introduction of alkaline nanosheets of LIC, the acidic degradation products of the hydrogel were effectively neutralized, the chemical‐humoral microenvironment was improved, and additional functional factors were introduced. As shown in Figure 2h, the pH value of the GA‐HBC‐LIC decomposition products approached physiological levels (7.0–7.4), contrasting starkly with the acidic pH of GA‐HBC hydrogel (6.1–6.3).^[^ ^19^ ^]^ Scanning electron microscopy (SEM) displayed that the internal pore size of the hydrogel was ≈50–200 µm, facilitating the exchange and circulation of chemicals within body fluids (Figure S1g, Supporting Information). The incorporation of LIC roughened the internal pore walls, conducive to cell adhesion, proliferation, and differentiation (Figure 2i).^[^ ^20^ ^]^ Elemental mapping and X‐ray diffraction (XRD) patterns confirmed the successful synthesized of GA‐HBC‐LIC hydrogel (Figure 2j; Figure S1h, Supporting Information).

### 2.2 Performance Evaluation of the Hydrogel System

**Figure 3:**
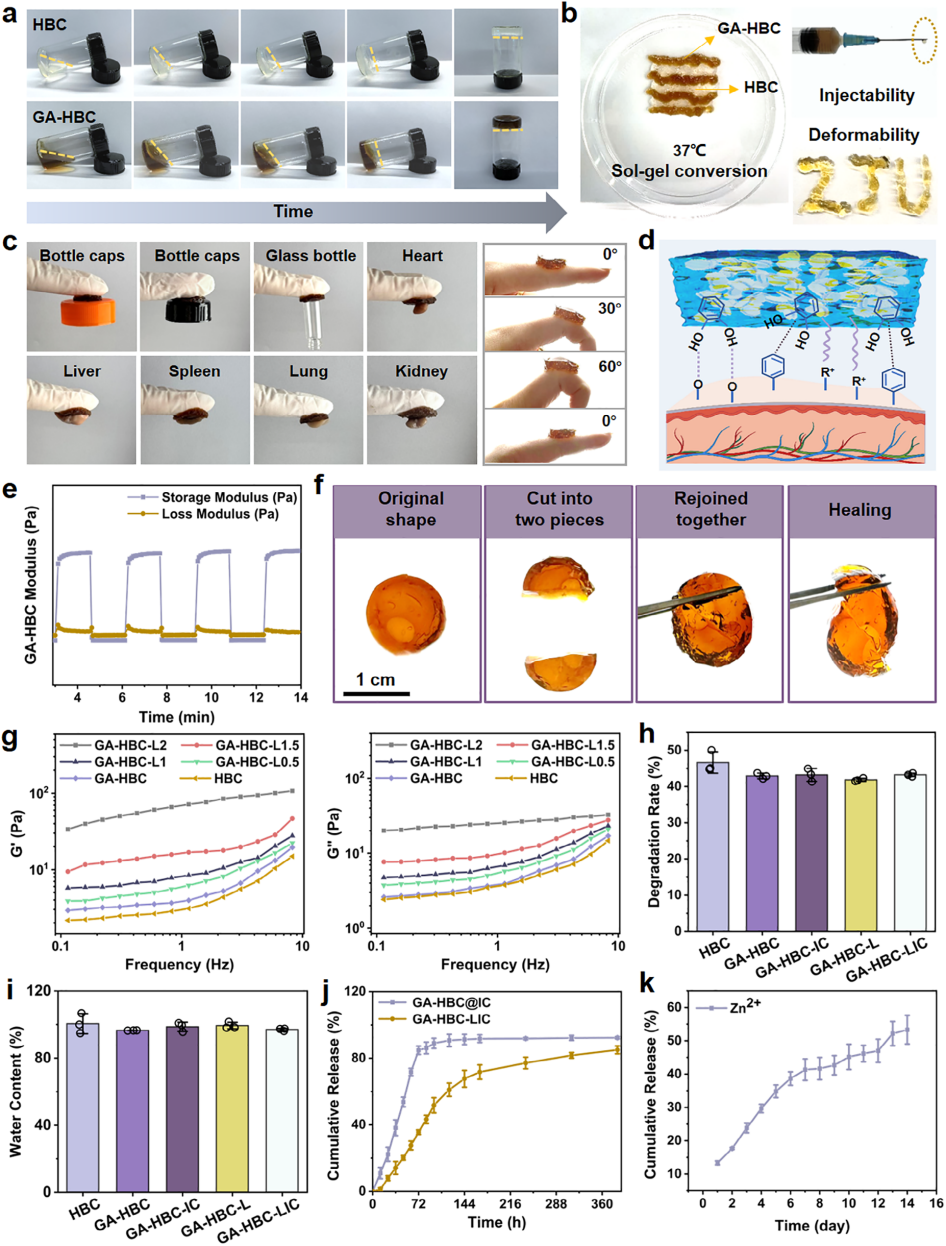
Performance evaluation of the hydrogel system. a) Diagram of HBC and GA‐HBC hydrogels gelation. b) Injectability of HBC and GA‐HBC hydrogels through the syringe. c) Adhesive capacity on various substrates and adhesion to rat organs (heart, liver, spleen, lung, and kidney). d) Adhesion mechanism of the GA‐HBC hydrogel. e) Step‐strain sweep of the GA‐HBC hydrogel. f) Macroscopic self‐healing property of the GA‐HBC hydrogel. g) Storage modulus (G′) and loss modulus (G″) of various hydrogels in rheological characterization. h) Degradation rate of various hydrogels. i) Equilibrium water content of different samples. j) ICA release profiles from GA‐HBC‐LIC and GA‐HBC@IC hydrogels. k) Zn^2+^ release kinetic from the GA‐HBC‐LIC hydrogel.

Injectable thermosensitive hydrogels can dynamically fill irregular spaces within the periodontal pocket, providing a favorable biomimetic microenvironment.^[^ ^21^ ^]^ HBC and GA‐HBC hydrogels can rapidly transition from sol to gel states at 37 °C (**Figure** **3**a; Figure S2a, Supporting Information). The hydrogel can be extruded effortlessly through a needle (0.5*16 mm) to form a specific pattern, such as “ZJU”, and transform into a gel‐like state at 37 °C in water, demonstrating excellent deformability (Figure 3b, Video S1, Supporting Information). Viscosity, stability, and mechanical strength are crucial for implantation and repair applications, such as tissue adhesion and cell attachment.^[^ ^22^ ^]^ Figure S2b,c (Supporting Information) showed that the average adhesion strength of GA‐HBC hydrogel (8.9 kPa) was significantly higher than that of HBC hydrogel (*p <* *0.05*; 3.32 kPa). Notably, GA‐HBC hydrogel can still adhere closely to various substances and tissues, maintaining stable during 0°–60° finger flexion and under running water flow (Figure 3c, Video S2, Supporting Information). Owing to the abundant hydroxyl, amino, and amide groups in GA‐HBC hydrogel that can form robust hydrogen bonds with tissue surface active groups (e.g., amino and hydroxyl), thereby enabling it to withstand the forces generated by regenerating tissues (Figure 3d).^[^ ^23^ ^]^ Cyclic strain experiments demonstrated that HBC and GA‐HBC hydrogels maintained structural integrity of their frameworks (Figure 3e; Figure S2d, Supporting Information).^[^ ^24^ ^]^ The fractured surfaces of both materials could recombine seamlessly at 37 °C (Figure 3f). Compared to HBC hydrogel, GA‐HBC hydrogel exhibited enhanced mechanical properties, allowing it to endure significantly higher levels of stress, thus showing greater potential for clinical application (*p <* *0.05*; Figure S3a,b, Supporting Information).

Subsequently, the effect of incorporating LDHs into GA‐HBC hydrogel on its rheological behavior was investigated (Table S2, Supporting Information). The shear‐thinning properties of the hydrogels were confirmed to validate their injectability (Figure S4a, Supporting Information). As the frequency increased, both G′ and G″ gradually rose and G' consistently remaining higher than G″, indicating excellent stability of the gel system (Figure 3g).^[^ ^25^ ^]^ The strain‐resistive performance of the hydrogels improved as the concentration of LDHs increased (Figure S4b, Supporting Information). Furthermore, all critical temperature values for the sol‐gel transition in these systems were within 37 °C, effectively responding to body temperature for solidification (Figure S5, Supporting Information). In conclusion, GA‐HBC hydrogel exhibited enhanced performance and further augmented with increasing concentrations of LDHs. However, to avert the damage inflicted on normal cell tissues, it is essential to determine the safe concentration of LDHs. The viability of cells in GA‐HBC‐L1 group was ≈90%, meeting the basic requirements for tissue engineering and enabling its application in subsequent experiments (Figure S6 and Table S3, Supporting Information).

Notably, biodegradability plays a crucial role in determining the material efficacy.^[^ ^26^ ^]^ Ideally, materials for periodontal regeneration should remain effective in the body for a minimum of 4–6 weeks.^[^ ^27^ ^]^ Rapid degradation may lead to the premature collapse of the bone cavity, while slow degradation may impede new tissue formation.^[^ ^28^ ^]^ At 14 days, no significant differences were observed in the degradation behavior among the groups, with a degradation rate of ≈44% (Figure 3h; Figure S7, Supporting Information). In vivo degradation characteristics of various hydrogels were accordingly evaluated. At various time points (0, 2, and 4 weeks), the hydrogels were well retained in situ (Figure S8a, Supporting Information). By 4 weeks, all samples exhibited significant degradation, with a degradation rate of ≈75% (*p <* *0.05*; Figure S8b, Supporting Information). Research on degradation has shown that the hydrogels follow a suitable degradation curve, effectively guiding and supporting the inward growth of new bone tissue. Each group of samples displayed the water content exceeding 90%, with a swelling index of nearly 4%, showcasing exceptional water absorption and retention properties (Figure 3i; Figure S9a, Supporting Information). These materials possess the ability to swiftly absorb wound exudate, thereby facilitating an optimal moist environment conducive to wound healing.^[^ ^29^ ^]^

Eventually, we assessed the release behavior of ICA in both hydrogels. The cumulative release rate of ICA in the GA‐HBC@LIC group reached nearly 84.78% within 72 h. In contrast, the GA‐HBC‐LIC hydrogel achieved a sustained and gradual release of ICA, with a cumulative release rate of ≈35.57% at 72 h and up to 85.03% at 384 h (Figure 3j). Moreover, Zn^2^⁺ was released from the GA‐HBC‐LIC hydrogel in a controlled and gradual manner, with cumulative release rates of ≈22% after 3 days and 42% after 7 days (Figure 3k; Figure S9b, Supporting Information). Therefore, the customized GA‐HBC‐LIC hydrogel system exhibited exceptional mechanical properties and biodegradability, enabling the effective establishment and maintenance of dynamic physical spaces within the periodontal pocket.^[^ ^30^ ^]^ The interaction between LIC and GA‐HBC facilitated the self‐release of Zn^2^⁺ and ICA, forming an ideal chemical‐humoral microenvironment for the development of subsequent physiological processes.

### 2.3 Bactericidal Effects of the GA‐HBC‐LIC Hydrogel In Vitro

**Figure 4:**
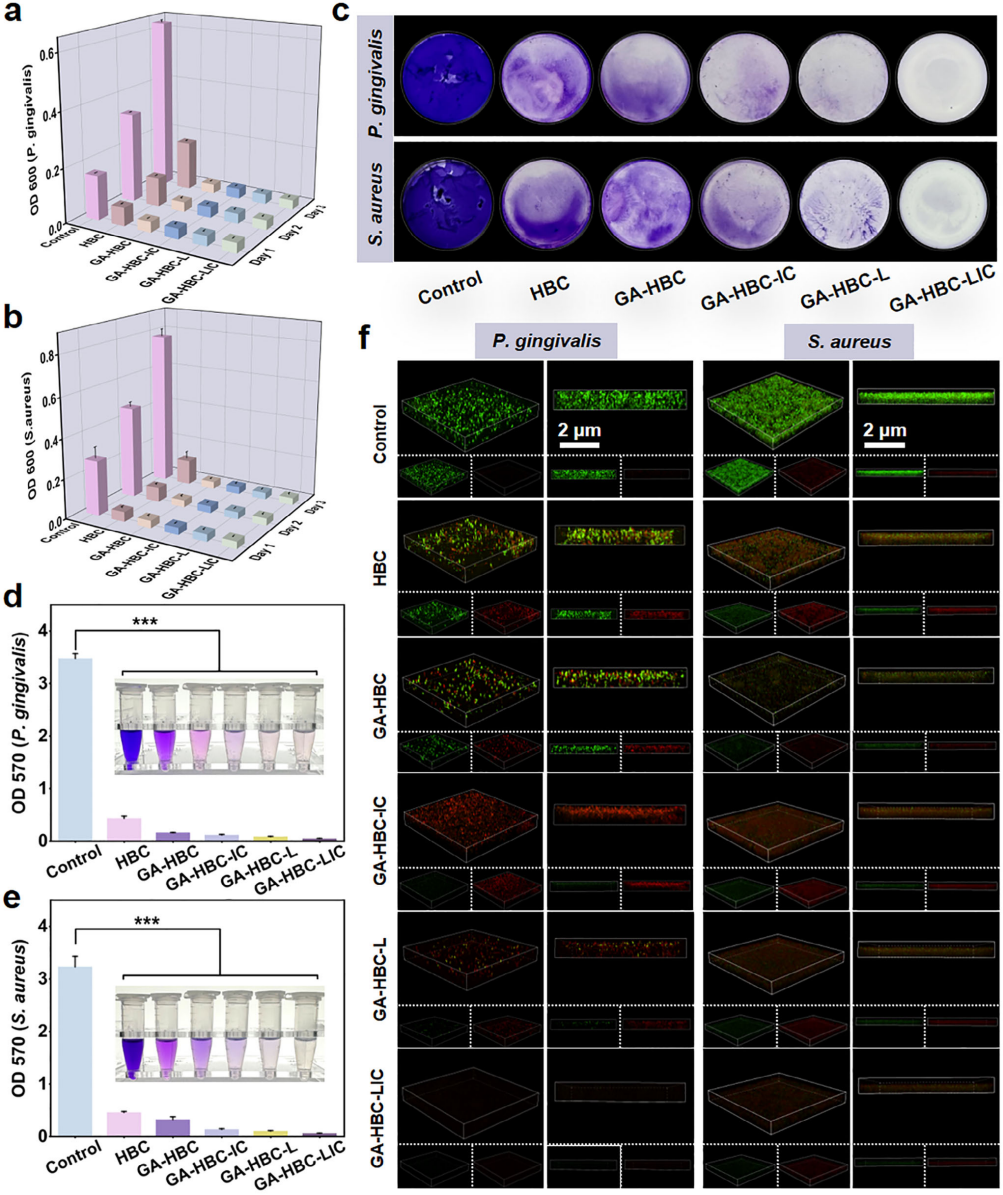
Bactericidal effects of the hydrogel system. Corresponding statistical analysis of *P. gingivalis* a) and Staphylococcus aureus (*S. aureus*, b) viability. c) Digital images of crystal violet‐stained biofilms of *P. gingivalis* and *S. aureus* after different treatments. Absorbances at 570 nm of crystal violet‐stained *P. gingivalis* d) and *S. aureus* e) biofilms (Illustration shows a photograph of the crystal violet staining quantitative experiment). f) Confocal fluorescence microscopy images of biofilms (comprising *P. gingivalis* and *S. aureus*) after different treatments, co‐stained with DMAO (green) and PI (red). ***p < *0.001*.

Given that the etiology of periodontitis is attributed to dental plaque, it is imperative to eradicate pathogenic bacteria and consistently suppress subsequent infections during the initial phase.^[^ ^31^ ^]^ In vitro antibacterial trials demonstrated that all GA‐HBC‐based groups exhibited antibacterial activities of ≈80% on day 1, increasing to nearly 95% by days 2–3 (**Figure** **4**a,b). The number of free bacteria in the HBC group was lower than that in the control group, while the GA‐HBC group almost completely inhibited bacterial colony formation, indicating the hydrogel's powerful capacity to entrap bacteria (Figure S10, Supporting Information). After that, damage to bacterial membranes was investigated using SEM. As shown in Figure S11 (Supporting Information), most bacteria in the experimental group were damaged, with surface collapse and wrinkling, cell protoplasm leakage and aggregation.^[^ ^32^ ^]^ The potent antibacterial properties of this compound can be attributed to three main factors. First, the cationic structure of CS in HBC interacts with the anionic components of the cell membrane, disrupting its integrity.^[^ ^33^ ^]^ Second, GA inhibits bacterial growth by degrading cellular morphology, increasing membrane permeability, and releasing intracellular components.^[^ ^34^ ^]^ Third, it suppresses biofilm formation by inhibiting the synthesis of proteins and polysaccharides in the biofilm structure.^[^ ^35^ ^]^ To mimic in vivo bacteria living microenvironment, anti‐biofilm capacity of the hydrogel was evaluated. Crystal violet staining and quantitative analysis revealed that both GA‐HBC‐L and GA‐HBC‐LIC hydrogels exhibited significant inhibitory effects on bacterial biofilm formation (*p <* *0.05*; Figure 4c–e). This enhanced efficacy can be attributed to the presence of Zn^2+^, as many studies have reported that Zn^2+^ can inhibit biofilm formation.^[^ ^36^ ^]^ As similarly presented in Figure 4f, the number of dead bacteria (red fluorescence) rapidly increased in biofilms treated with hydrogels. Simultaneously, biofilm formation in the GA‐HBC‐L and GA‐HBC‐LIC groups was significantly reduced, with almost no viable bacteria (*p <* *0.05*; green fluorescence). Collectively, these findings indicated that GA‐HBC‐LIC hydrogel is a potent bactericide with significant antibacterial application potential. Specifically, the synergistic interaction between GA, CS, and Zn^2^⁺ effectively eliminates persistent bacterial colonies in periodontal pockets and on root surfaces, thereby preventing infection recurrence.^[^ ^37^ ^]^

### 2.4 Biocompatibility Assessment and Osteogenic Study In Vitro

In light of the growing need for clinical tissue restoration and rejuvenation, impeccable biocompatibility is a crucial prerequisite for biomaterials.^[^ ^38^ ^]^ Fluorescent images of all groups clearly demonstrated the absence of dead cells (Figure S12a, Supporting Information). Cell counting kit‐8‌ (CCK8) assays confirmed the negligible cytotoxicity of the hydrogels, with cell viability approaching 100% in all groups (Figure S12b, Supporting Information). Given the direct contact of hydrogels with tissues, hemolysis tests revealed that none of the samples induced significant hemolysis (Figure S12c, Supporting Information).^[^ ^39^ ^]^ Overall, the GA‐HBC‐LIC hydrogel exhibited exceptional biocompatibility and non‐hemolytic properties, rendering it a strong candidate for biomedical use.

**Figure 5:**
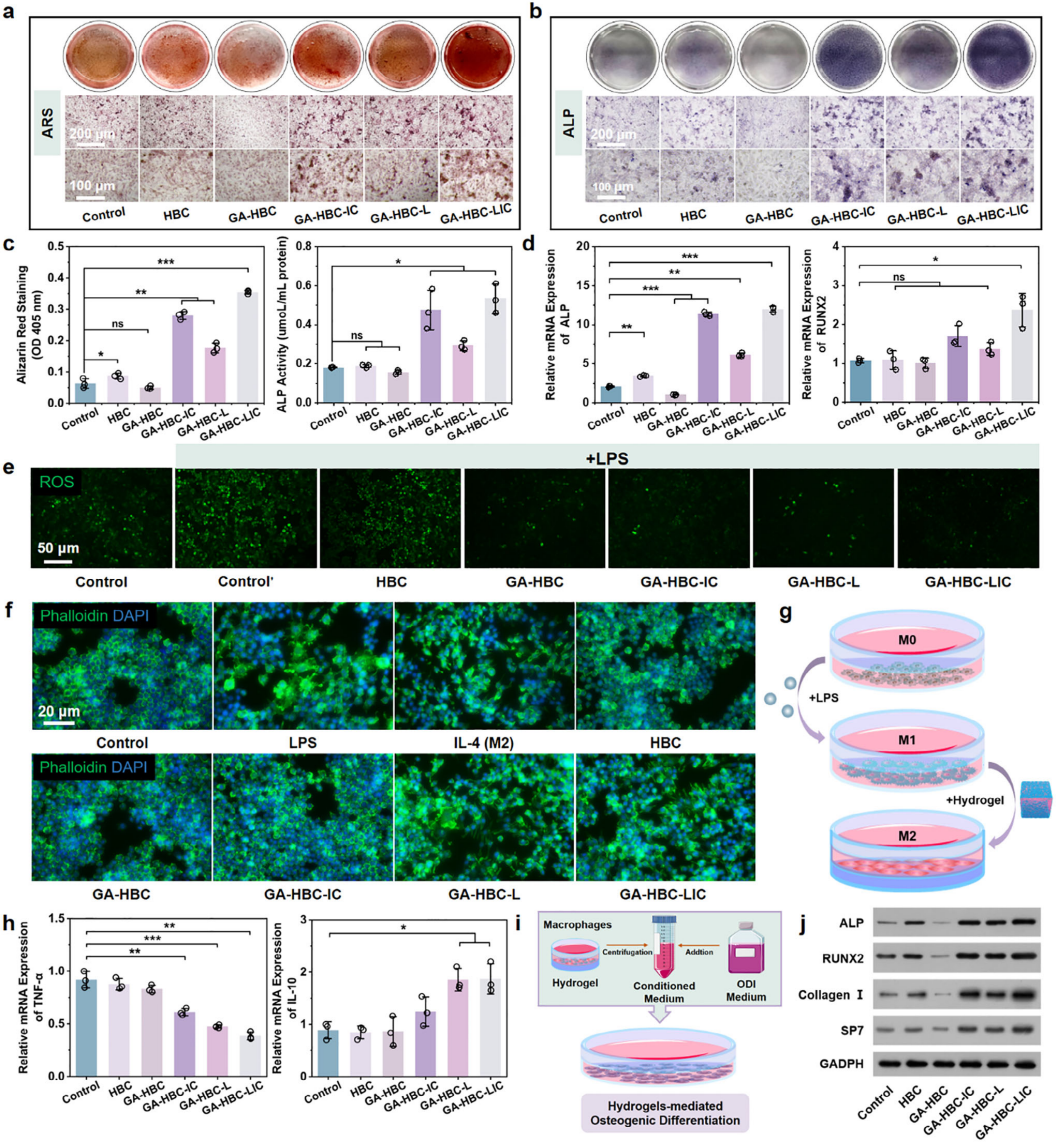
Anti‐inflammatory and osteogenic performance study. Alizarin red staining (ARS) images a) and alkaline phosphatase (ALP) staining images b) of MC3T3 cells co‐cultured with various hydrogels for 14 days. c) Corresponding quantitative red areas and quantitative ALP activity at day 14 of MC3T3 cells co‐cultured with various hydrogels. d) Relative mRNA expression of osteogenesis‐related genes at 14 days. e) Fluorescent microscopy images of Raw264.7 cells labeled with DCFH. f) Cytoskeletal staining images of Raw264.7 cells cultured under standard conditions or co‐cultured on hydrogels for 24 h. g) Illustrative diagram of hydrogel‐induced macrophage polarization. h) Relative mRNA expression of inflammation‐related genes. i) Illustrative diagram of MC3T3 cells differentiation induced by conditioned medium. j) Protein expression levels of ALP, SP7, RUNX2, and Collagen I in MC3T3 cells determined by Western blot. *p < 0.05, **p < 0.01, ***p < *0.001*.

The ideal hydrogel for bone repair is supposed to possess the osteoinductive function.^[^ ^40^ ^]^ Therefore, we investigated the potential osteogenic ability of these hydrogels. Indeed, cells underwent rapid proliferation and growth within a short period, which is conducive to subsequent osteogenic mineralization (Figure S13, Supporting Information).^[^ ^41^ ^]^ After 14 days of culture, the alkaline phosphatase (ALP) activity was higher and calcium nodule formation was more pronounced in the GA‐HBC‐LIC group, consistent with the quantitative findings, suggesting the synergistic osteogenic effect of the LDHs and ICA (**Figure** **5**a–c). Additionally, the expression levels of osteogenesis‐related genes (*ALP*, runt‐related transcription factor 2 (*RUNX2*), osterix (*SP7*), and *Collagen I*) in the GA‐HBC‐LIC group were the highest, demonstrating the extra osteogenic property from the sustained release of ICA (Figure 5d; Figure S14a, Supporting Information). In conclusion, the GA‐HBC‐LIC hydrogel provided an exceptional physicochemical spatial environment, exhibiting remarkable osteogenic potential in vitro.

### 2.5 Coupling of Immunomodulation and Osteogenesis In Vitro

Looking back on our findings, we have concluded that the GA‐HBC‐LIC hydrogel possesses satisfying antibacterial and osteogenic properties. Although effective antibacterial treatment can mitigate inflammation, persistent redox imbalance and immune suppression—namely, a chemically imbalanced humoral milieu—can significantly impair osteogenic capacity.^[^ ^42^ ^]^ Therefore, whether an injectable customized GA‐HBC‐LIC hydrogel system can modulate the inflammation‐immune microenvironment remains a challenge. GA, a natural antioxidant, was evaluated for its reactive oxygen species (ROS)‐scavenging ability in hydrogels.^[^ ^43^ ^]^ As depicted in Figure S14b–d (Supporting Information), the scavenging rates of 1,1‐Diphenyl‐2‐picrylhydrazyl (DPPH), hydrogen peroxide (H_2_O_2_), and hydroxyl radical (·OH) by the GA‐HBC hydrogel were ≈79.7%, 65.2%, and 60%, respectively, demonstrating its effective free radical scavenging capacity. Consistent with expectations, the fluorescence intensity of GA‐based hydrogels was significantly reduced compared to lipopolysaccharide (LPS) exposure (*p <* *0.05*; Figure 5e; Figure S14e, Supporting Information). Thus, the utilization of hybrid hydrogels in therapy can effectively eliminate exogenous oxidants and stimulate endogenous antioxidant activity, thereby enhancing macrophages' resistance to oxidative stress and facilitating the resolution of inflammation.

Macrophages are among the most crucial immune cells, capable of exhibiting distinct polarization states, including the pro‐inflammatory (M1) phenotype and the anti‐inflammatory (M2) phenotype.^[^ ^44^ ^]^ In periodontitis, the phenotypic transition of macrophages from M1 to M2 is impaired, leading to chronic inflammation and immune system dysfunction.^[^ ^45^ ^]^ Considering that we have developed a customized hydrogel system capable of self‐triggered acid response and sustained release of Zn^2^⁺, which can effectively reduce macrophage M1 polarization and induce M2 polarization.^[^ ^46^ ^]^ We postulated that the anti‐inflammatory and immunomodulatory effects of the GA‐HBC‐LIC hydrogel are intricately linked to the modulation of macrophage phenotype mediated by Zn^2^⁺. To substantiate this hypothesis, we employed phalloidin staining to examine changes in macrophage morphology. As shown in Figure 5f, macrophages exposed to GA‐HBC‐L and GA‐HBC‐LIC hydrogels adopted spindle‐shaped morphologies, similar to the IL‐4 induced M2 phenotype, while the control group remained small and round. Zn^2+^ staining indicated higher fluorescence intensity in LDHs‐containing hydrogels (Figure S14f, Supporting Information). Combined with the results of macrophage morphological changes, it suggested that macrophage phenotypic transformation occurred under the action of Zn^2+^. The expression alterations of inflammation‐related genes in macrophages after diverse treatments were analyzed via qRT‐PCR, indicating the downregulation of inflammatory factors (tumor Necrosis Factor‐α (*TNF‐α*) and interleukin‐1 beta (*IL‐1β*))and upregulation of anti‐inflammatory factors (interleukin‐10 (*IL‐10*) and interleukin‐4 (*IL‐4*)) in the GA‐HBC‐L and GA‐HBC‐LIC groups (Figure 5g,h; Figure S15a, Supporting Information). This further confirmed the polarization state of macrophages. Having established the immunomodulatory role of the hydrogels on macrophages, we then explored their consequent impact on osteogenic differentiation. MC3T3 cells were cultured in specialized growth medium (Figure 5i). Western blotting of ALP, RUNX2, SP7, and Collagen I proteins demonstrated significant upregulation in the GA‐HBC‐IC, GA‐HBC‐L, and GA‐HBC‐LIC groups, with the GA‐HBC‐LIC group showing the most substantial increase (*p <* *0.05;* Figure 5j; Figure S15b, Supporting Information). Therefore, the release of Zn^2+^ during degradation effectively shifted macrophages from M1 to M2 phenotype, synergizing with GA's antioxidant properties to combat inflammation and modulate immune responses, creating an optimal chemical balanced humoral milieu for the ICA to exert its osteogenic function, resulting in a combined effect greater than the sum of its parts.

### 2.6 Impact of the GA‐HBC‐LIC Hydrogel on Reducing Inflammatory Bone Loss In Vivo

During bone remodeling, the dynamic balance between osteoblasts and osteoclasts maintains the integrity of bone structure.^[^ ^47^ ^]^ Bacterial infection‐induced oxidative stress and excessive inflammatory responses in periodontal pockets result in persistently elevated osteoclast activity, which impairs functional bone repair and hinders tissue healing.^[^ ^48^ ^]^ Given the promising results from in vitro experiments, it is essential to further investigate whether the GA‐HBC‐LIC hydrogel can translate its beneficial properties into an in vivo setting and exert sustained therapeutic effects over time.

**Figure 6:**
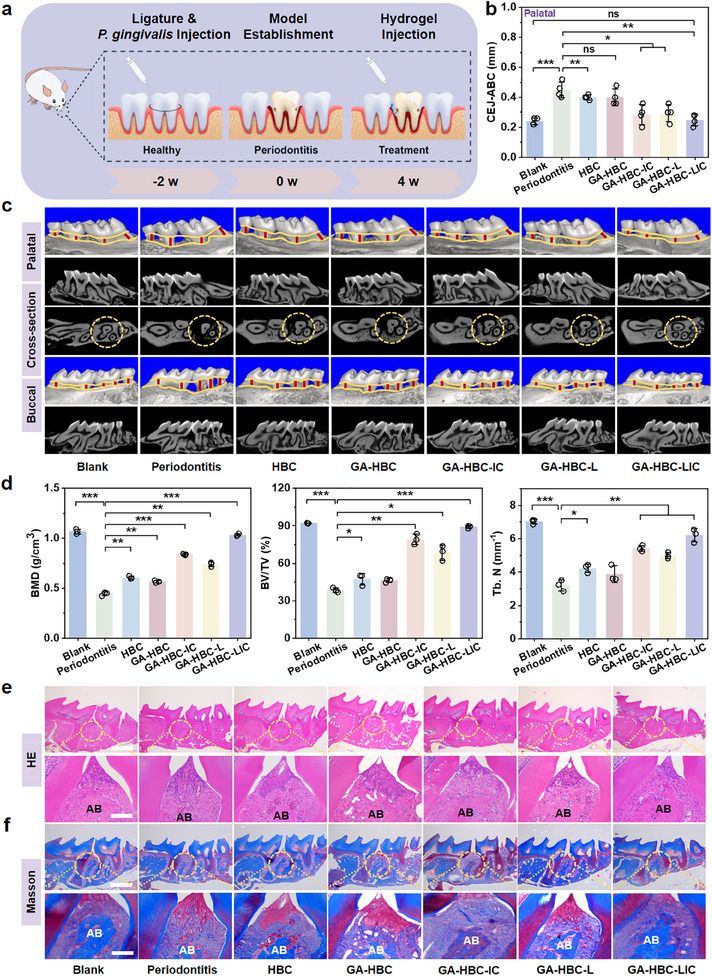
In vivo study. a) Schematic illustration of the establishment of the periodontitis model followed by hydrogels injection. b) Quantitative analysis of palatal bone resorption and bone formation (CEJ‐ABC) in each group. c) Micro‐CT reconstruction images of maxillary bone tissue in each group. d) Bone volume/total volume (BV/TV), bone mineral density (BMD), and trabecular number (Tb. N) were assessed using Micro‐CT quantitative analysis. e) H&E staining images of periodontal tissues in different groups (scale bar: 200 and 50 µm). f) Masson‐trichrome staining images of periodontal tissues in different groups (scale bar: 200 and 50 µm). *p < 0.05, **p < 0.01, ***p < *0.001*.

Initially, the in vivo biological safety of the hydrogels was demonstrated through histological evaluation of major organs and blood analysis (Figures S16 and S17, Supporting Information).^[^ ^49^ ^]^ In the experimental periodontitis model, after 4 weeks of treatment, the cement‐to‐enamel junction to alveolar bone crest (CEJ‐ABC) distances on the buccal and palatal sides of the GA‐HBC‐LIC group were remarkably reduced to 0.24 and 0.25 mm, respectively (*p < 0.05*; **Figure** **6**a,b; Figure S18a, Supporting Information). Micro‐computed tomography (Micro‐CT) images and quantitative analyses revealed effective bone regeneration at the alveolar crest in the GA‐HBC‐LIC group compared to the HBC and GA‐HBC groups, with notable improvements in bone‐related parameters (*p < 0.05*; Figure 6c,d; Figure S18b, Supporting Information). Additionally, compared to the periodontitis group, the GA‐HBC‐LIC group exhibited a significant reduction in inflammatory cell infiltration, orderly arrangement of collagen fibers (stained blue), and partial restoration of periodontal tissue, similar to the blank group (*p <* *0.05*; Figure 6e,f). Likewise, the expression of factors (e.g., osteopontin (OPN), bone morphogenetic protein type 2 (BMP‐2), and vascular endothelial growth factor (VEGF)) associated with tissue healing was noticeably increased in the GA‐HBC‐LIC group (*p <* *0.05*; Figure S19, Supporting Information). Macrophages are key players in the local immune response during tissue damage.^[^ ^7^, ^50^ ^]^ Reducing M1 macrophages and enhancing M2 macrophages can facilitate effective wound healing.^[^ ^51^ ^]^ Consistent with in vitro findings, the fluorescence of inducible nitric oxide synthase (iNOS), a M1 biomarker, was nearly undetectable in the GA‐HBC‐L and GA‐HBC‐LIC groups, while the fluorescence intensity of CD206, a M2 biomarker, was markedly elevated (Figure S20, Supporting Information).

In summary, we achieved bone immune regulation by incorporating alkaline Zn^2+^‐based LIC nanosheets into the GA‐HBC hydrogel, which possesses excellent antibacterial and antioxidant properties. This approach effectively addressed the contradiction between rapid degradation and avoiding excessive toxicity. The addition of 0.01% (wt/vol) LDHs provided a robust mechanical foundation for the scaffold, ensuring spatial stability. The simultaneous release of GA and Zn^2+^ from the GA‐HBC‐LIC hydrogel significantly enhanced the polarization of macrophages toward anti‐inflammatory phenotype, facilitating the transition from the inflammation stage to the repair stage. Furthermore, the in situ continuous release of ICA, coupled with appropriate material degradation and the formation of a bone immune microenvironment, facilitated the replacement of newly formed bone tissue within the dynamic physical space. This process significantly increased the levels of bone formation markers, enhanced collagen deposition, and promoted new bone formation.

### 2.7 Sequential Immune Modulation of the GA‐HBC‐LIC Hydrogel Propelled by ZEB1 In Vitro

**Figure 7:**
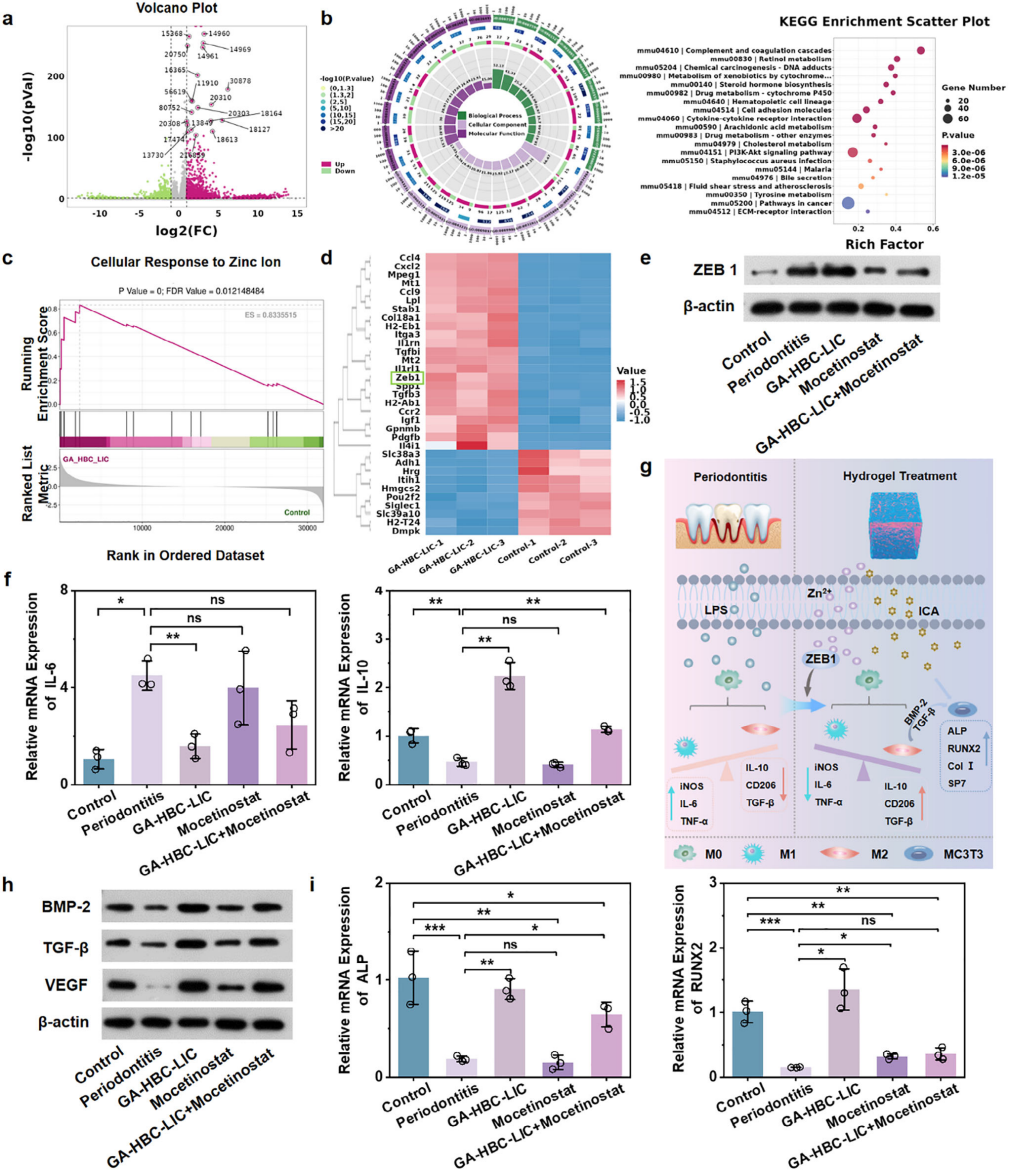
Sequential immune modulation of the GA‐HBC‐LIC hydrogel in vitro. a) Volcano plot of the differentially expressed genes identified by RNA‐seq. b) Go analyses and KEGG pathway enrichment comparing the GA‐HBC‐LIC group with the control group. c) GSEA of pathways following RNA‐seq. d) Heat map depicting the expression patterns of 33 regulated genes. e) Protein expression level of ZEB1 in Raw264.7 cells determined by Western blot. f) Relative mRNA expression of inflammation‐related genes. g) Schematic illustration of the therapeutic mechanism of GA‐HBC‐LIC hydrogel under periodontitis microenvironment. h) Western blot analysis of BMP‐2, TGF‐β, and VEGF protein expression levels in MC3T3 cells. i) Relative mRNA expression levels of osteogenesis‐related genes at 14 days. *p < 0.05, **p < 0.01, ***p < *0.001*.

To profoundly elucidate the immune regulatory mechanism and potential biological functions of the GA‐HBC‐LIC hydrogel, this study employed RNA‐sequencing (RNA‐seq) technology to investigate the transcriptional alterations in macrophages following diverse treatments. Principal component analysis (PCA) identified 1711 significantly differentially expressed genes (DEGs) in the GA‐HBC‐LIC group, with 504 genes downregulated and 1207 genes upregulated (**Figure** **7**a; Figure S21, Supporting Information). Gene Ontology (GO) analyses and Kyoto Encyclopedia of Genes and Genomes (KEGG) pathway enrichment revealed that the enriched biological processes primarily focused on biological adhesion, inflammation, and immune‐related processes (Figure 7b). Gene Set Enrichment Analysis (GSEA) unveiled that the signaling pathways related to inflammation, bone formation, and cell migration/adhesion exhibited an upward trend (Figure S22, Supporting Information). Of note, Zn^2+^‐mediated cellular responses were significantly enhanced (Figure 7c). As previously mentioned, Zn^2^⁺ is crucial for macrophage polarization and immune regulation, although its specific mechanisms remain unexplored. Through the analysis of the DEGs heat map, it was discovered that the ZEB1 gene related to Zn^2+^ was highly expressed in the GA‐HBC‐LIC group (Figure 7d). Existing studies have shown that Zn^2+^ can activate the transcription factor function of ZEB1 by binding to its zinc finger domain.^[^ ^52^ ^]^ GA‐HBC‐LIC hydrogel treatment significantly upregulated the expression of ZEB1, while it is significantly suppressed after treated with the ZEB1‐targeted inhibitor, Mosetinostat, confirming Zn^2+^’s effect on the ZEB1 target (*p < 0.05*; Figure 7e; Figure S23, Supporting Information).^[^ ^53^ ^]^ However, in the LPS‐treated group, ZEB1 expression was significantly elevated compared to the control group. This could potentially be explained by the acute inflammation induced by LPS, which resulted in reduced miR‐200c levels.^[^ ^54^ ^]^ It has been reported that ZEB1 regulates macrophage inflammation and immunosuppression and is crucial for metformin's anti‐inflammatory and ROS‐inhibiting effects in both acute and chronic inflammation models such as psoriasis.^[^ ^55^ ^]^ We thus postulated that GA‐HBC‐LIC's smartly released Zn^2+^ promotes macrophage phenotype transformation from M1 to M2 by upregulating ZEB1. As illustrated in Figure 7f, the pro‐inflammatory cytokine interleukin‐6 (*IL‐6*) was significantly downregulated in the GA‐HBC‐LIC group, while the anti‐inflammatory cytokine *IL‐10* was upregulated, with no significant difference observed in the Mosetinostat group (*p < 0.05*). These findings collectively indicated that Zn^2+^‐mediated macrophage polarization can be significantly suppressed by Mosetinostat, further elucidating that its immune regulation process is primarily achieved through the ZEB1 target. Consistent with our data, studies have shown that ZEB1 deletion delays macrophage anti‐inflammatory phenotype transformation, exacerbating muscle injury and impeding regeneration in inflammatory settings.^[^ ^56^ ^]^ Inhibition of miR‐200c and facilitation of the ZEB1/Notch signaling pathway regulate secreted osteogenic factors and favor mineralization under oxidative stress conditions.^[^ ^54^, ^57^ ^]^ Thus, Zn^2+^‐mediated transformation of macrophages from M1 to M2 phenotype via the ZEB1 target not only regulates chronic inflammation but also promotes the secretion of cytokines (e.g., transforming growth factor‐β (TGF‐β), BMP‐2, and VEGF), and the proliferation and differentiation of osteoblasts, confirming its dual role in inflammation alleviation and tissue repair (Figure 7g–i). Therefore, it is anticipated that the GA‐HBC‐LIC hydrogel can optimize the chemical‐humoral space within the periodontal pocket via ZEB1, thereby achieving an ordered regulation of the inflammation elimination and tissue regeneration processes, and effectively preventing alveolar bone loss and mitigating periodontitis.

### 2.8 Spatiotemporal Therapeutic Effects of the GA‐HBC‐LIC Hydrogel in a ZEB1‐Inhibited Periodontitis Mouse Model

**Figure 8:**
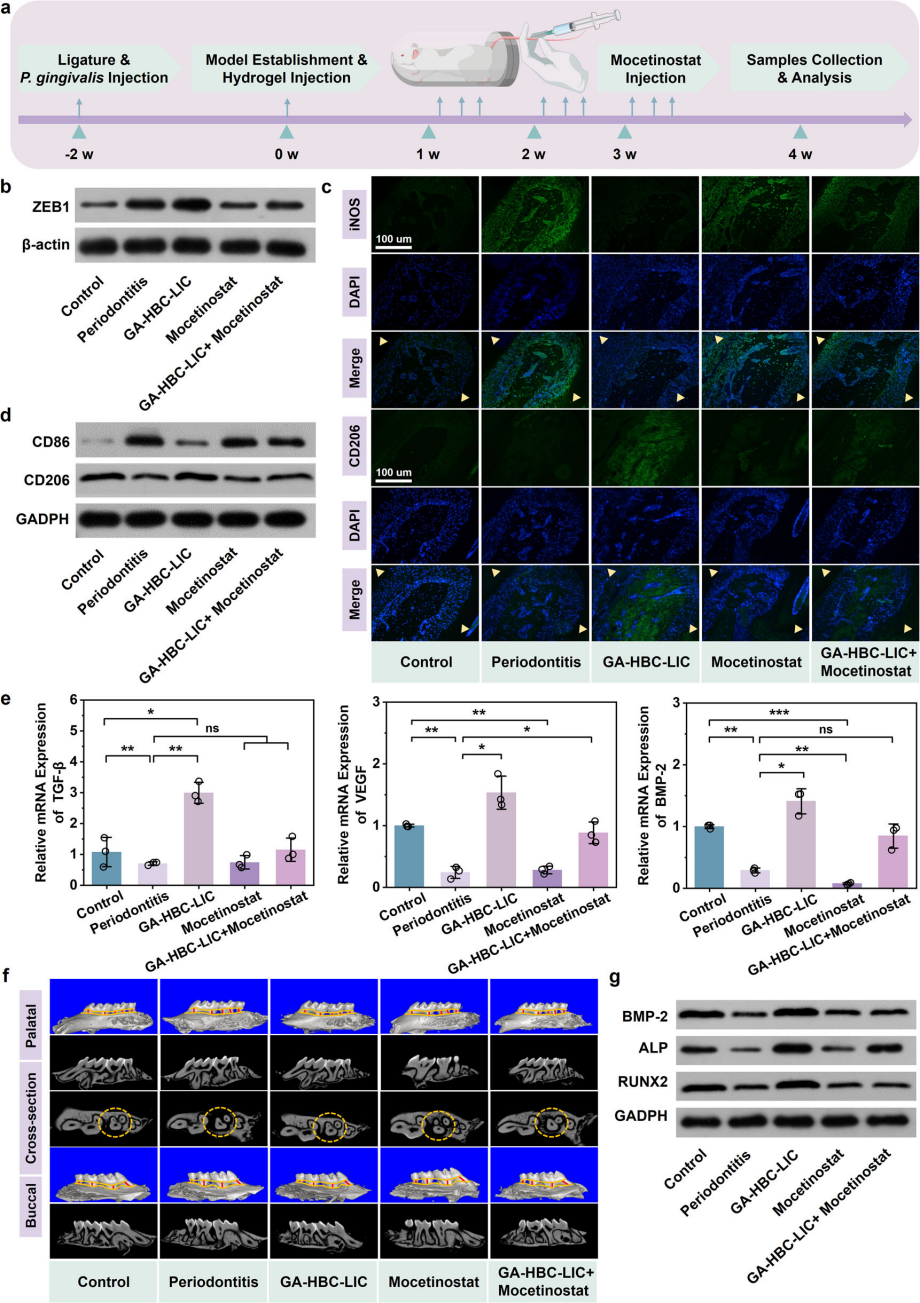
Spatiotemporal therapeutic effects of the GA‐HBC‐LIC hydrogel in vivo. a) Schematic illustration of the periodontitis model with Mocetinostat treatment. b) Western blot analysis of ZEB1 protein expression levels in maxillary tissues. c) Immunofluorescence staining of iNOS and CD206 in maxillary tissues following different treatments. d) Western blot analysis of CD86 and CD206 protein expression levels in maxillary tissues. e) Relative mRNA expression levels of *TGF‐β*, *VEGF*, and *BMP‐2* cytokines in maxillary tissues following different treatments. f) Micro‐CT reconstruction images of bone tissue in each experimental group. g) Western blot analysis of BMP‐2, ALP, and RUNX2 protein expression levels in maxillary tissues. *p < 0.05, **p < 0.01, ***p < *0.001*.

To validate the in vivo efficacy of the Zn^2^⁺‐mediated ZEB1 target, we established a mouse periodontitis model and administered Mocetinostat, a specific inhibitor of the Zn^2^⁺‐ZEB1 axis (**Figure** **8**a). In maxillary tissues, ZEB1 protein expression was significantly upregulated in the GA‐HBC‐LIC group, while Mocetinostat effectively inhibited this process, demonstrating its potent inhibitory effect on ZEB1 (*p <* *0.05*; Figure 8b). Furthermore, ZEB1 inhibition impeded the Zn^2^⁺‐mediated in vivo transition of macrophages from M1 to M2 phenotype, characterized by increased levels of pro‐inflammatory cytokines and significant downregulation of anti‐inflammatory factors, leading to sustained inflammation (*p < 0.05*; Figure 8c,d; Figure S24, Supporting Information). The aforementioned experimental results indicated that under the action of Zn^2^⁺, effective macrophage polarization creates a favorable chemical environment conducive to osteoinduction, promoting the regeneration of functional periodontal tissues. Therefore, evaluation of osteogenesis‐related processes revealed that compared to the GA‐HBC‐LIC group, the expression levels of *TGF‐β*, *BMP‐2*, and *VEGF* were significantly reduced in the GA‐HBC‐LIC + Mocetinostat group (*p <* *0.05*; Figure 8e). Micro‐CT imaging and histological analysis showed limited new bone tissue regeneration in the GA‐HBC‐LIC + Mocetinostat group due to the presence of the ZEB1 inhibitor, with no improvement in bone‐related parameters, indicating that the impaired improvement of inflammatory microenvironment (Figure 8f,g; Figures S25 and S26, Supporting Information). In conclusion, ZEB1 is the central target of Zn^2^⁺ action, playing a pivotal role in the treatment of chronic periodontitis and serving as the key to unlocking the anti‐inflammatory and tissue repair functions of the GA‐HBC‐LIC hydrogel.

Reviewing the past, we ingeniously designed a customized hydrogel system of GA‐HBC‐LIC with temporal and spatial sequence therapeutic properties, by integrating the physical support space and the chemically modified interstitial fluid space into the sol‐gel transition formulation. We have found that the application of the temperature‐sensitive hydrogel system with outstanding mechanical properties to the tissue defect sites can form an isolated dynamic physical space. Herein, Zn^2^⁺ played a pivotal role throughout the space by orchestrating a cascade of events that link past and future. Initially, Zn^2^⁺ could exert a synergistic effect with GA, functioning as a scavenger for pathogenic bacteria and excessive ROS, possessing efficient antibacterial and antioxidant capabilities. Subsequently, the Zn^2^⁺‐mediated ZEB1 target achieved immune regulation of macrophages, eliminating various obstacles for the action of ICA and presenting a satisfactory positive effect in bone formation. Through an in‐depth investigation of the immune cell‐driven tissue healing process, we identified ZEB1 as a critical target. Subsequent reverse experiments confirmed the pivotal role of the Zn^2^⁺‐ZEB1 axis in coupling immune regulation with bone regeneration via the GA‐HBC‐LIC hydrogel. It governed the sequential occurrence of anti‐inflammation, immune regulation, and bone remodeling, thereby precisely embodying the quintessence of osteoimmunomodulation.

## 3 Conclusion

In this study, we have developed a customized injectable thermosensitive hydrogel system named GA‐HBC‐LIC, aimed at promoting the healing of chronic periodontitis with bone defects through a programmed spatiotemporal dynamic sequence therapy. In the early stage of treatment, the hydrogel can tightly and securely hold within the periodontal pocket, providing long‐lasting physical space dynamic support, while its components work synergistically to achieve short‐term bactericidal effects and inhibit infection. Subsequently, GA exhibited its antioxidant function, eliminating excess ROS in the pocket to promote the restoration of oxidative stress balance. Meanwhile, Zn^2+^ induced macrophage polarization from the M1 (pro‐inflammatory) to the M2 (anti‐inflammatory) phenotype by targeting ZEB1, regulating immune homeostasis. Ultimately, through the establishment of a favorable chemical microenvironment, the synergistic effect of ICA effectively promoted tissue regeneration and repair. Overall, our work takes periodontitis as an entry point, offering an alternative promising strategy for the treatment of other inflammatory diseases.

## 4 Experimental Section

### Chemical and Reagents

Zinc nitrate hexahydrate (Zn (NO_3_)_2_·6H_2_O, 99%), glacial acetic acid, sodium chloride, sodium hydroxide, and hydrogen peroxide (H_2_O_2_, 30%) were obtained from Sinopharm Chemical Reagent Co., Ltd. (MO, USA). Aluminum nitrate nonahydrate (Al (NO_3_)_3_·9H_2_O, 99%), ethanol (95%), and icariin (ICA, 97%) were purchased from Aladdin (Shanghai, China). 1‐ethyl‐(3‐dimethylaminopropyl) carbodiimide (EDC), N‐hydroxysuccinimide (NHS), and sodium β‐glycerophosphate were purchased from Macklin Biochemical Co., Ltd. (Shanghai, China). Lipopolysaccharide (LPS) and Trizol were purchased from Invivogen (CA, USA). 2,7‐Dichlorodihydrofluorescein diacetate (DCFH‐DA, ≥97%), dialysis tubes (8000—14 000 MWCO), phosphate buffered saline (PBS, pH  =  7.4), and L‐ascorbic acid were purchased from Sigma–Aldrich. Fetal bovine serum (FBS) was purchased from Lian Shuo Biotechnology Co., Ltd. (Shanghai, China). Cell Counting Kit 8 (CCK8) was purchased from Meilun Biotechnology Co., Ltd. FITC‐phalloidin was purchased from Yeasen Biotechnology Co., Ltd. (Shanghai, China). DPPH Free Radical Scavenging Capacity Assay Kit, Brain Heart Infusion Broth (BHI), gallic acid (GA, ≥98%), and chitosan (CS) were purchased from Beijing Solarbio Science & Technology Co., Ltd. Calcein AM, propidium iodide, BCIP/NBT alkaline phosphatase (ALP) chromogenic Kit, Alizarin Red S (ARS) staining kit, Hydrogen Peroxide Assay Kit, Live & Dead Bacterial Staining Kit, reactive oxygen species assay kit, Triton X‐100, 4′,6‐diamidino‐2‐phenylindole (DAPI), penicillin−streptomycin solution, and dexamethasone (≥99%) were obtained from Beyotime (Shanghai, China). Alkaline Phosphatase (AKP/ALP) Activity Assay Kit and Hydroxyl Radical Assay Kit were purchased from Beijing Boxbio Science & Technology Co., Ltd. Mouse Anti‐ZEB1 antibody was purchased from Bioss (USA). All the other antibodies were purchased from Elabscience (USA).

### Synthesis of ZnAl‐LDHs

ZnAl‐layered double hydroxide (ZnAl‐LDHs) nanosheets were synthesized via the co‐precipitation method.^[^ ^58^ ^]^ Zn(NO₃)₂·6H₂O (0.3 mol) and Al(NO₃)₃·9H₂O (0.1 mol) were separately added dropwise to vigorously stirred deionized (DI) water, while NaOH (0.4 mol) was continuously added to maintain the pH at 9–10. After completing the addition, the resultant turbid suspension was transferred to a hydrothermal autoclave and maintained at 80 °C for 24 h. Following ultrasonic treatment, centrifugation, and filtration, a water‐based dispersion of LDHs was obtained.

### Preparation of Hydrogels

1.0 g of CS was subjected to alkalization using a 10 mL solution of 50% NaOH (ω/ω) at room temperature for 24 h under N_2_ atmosphere. Subsequently, the alkalinized chitosan was filtered by centrifugation, and then dispersed in 20 mL of 50% (v/v) isopropanol for 30 min. Following this, 20 mL of oxidized butene was added to a round‐bottom flask and stirred at 55 °C for 24 h. The reacted solution was subsequently dialyzed (8000—14 000 MWCO) against deionized (DI) water for 5 days, followed by freeze‐drying to obtain hydroxybutyl chitosan (HBC). Then, 0.2 g HBC was dissolved in 10 mL DI water and then the pH was adjusted to 4–5. EDC·HCl (0.33 g) and NHS (0.198 g) were dissolved in 20 mL of ethanol (50 v/v%), followed by the addition of GA (0.292 g) for carboxylation. The resulting mixture was then combined with the HBC solution and gently agitated in the dark at room temperature for 24 h (N_2_ atmosphere). Finally, subject the solution to dialysis in a pH‐adjusted water ≈4.0–5.0, while avoiding light exposure for 5 days, followed by freeze‐drying to obtain GA‐HBC. A series of composite hydrogels were synthesized by incorporating varying amounts (0.005, 0.01, 0.015, and 0.02 wt/vol%) of LDHs into GA‐HBC, denoted as GA‐HBC‐L0.5, GA‐HBC‐L1, GA‐HBC‐L1.5, and GA‐HBC‐L2. Subsequently, the ICA‐modified LDHs were integrated into the GA‐HBC to yield the GA‐HBC‐LIC hybrid hydrogel.

### Materials Characterization

Transmission electron microscopy (TEM, Talos F200X, Thermo Scientific) was used at an accelerating voltage of 120 kV to examine the morphology of LDHs. High‐resolution transmission electron microscopy (HR‐TEM, Talos F200X, Thermo Scientific) was employed for detailed analysis of the lattice structure of LDHs. High‐angle annular dark field images and element mapping were obtained using a scanning transmission electron microscope (STEM, Talos F200X S with Super‐XTM, Thermo Scientific). The elemental composition of LDHs and the concentration of Zn^2+^ were determined via inductively coupled plasma atomic emission spectroscopy (ICP‐AES, Agilent ICP‐OES 5110). The particle size distribution of LDHs in different solutions (water and DMEM) as well as the zeta potentials of LDHs, ICA, and LIC were measured using dynamic light scattering (DLS, Zetasizer nano ZS90). Surface element analysis of LDHs were performed through X‐ray photoelectron spectroscopy (XPS, 250Xi, Thermo Scientific). The characteristic absorption peaks of ICA were evaluated using an UV‐visible spectrophotometer (UV‐2600, Shimadzu) to assess ICA loading efficiency and release profiles. The chemical structure of hydrogels was analyzed using Fourier Transform Infrared Spectroscopy (FT‐IR, Is50, Thermo Scientific). The freeze‐dried hydrogels were gold‐coated via sputtering and visualized with a scanning electron microscope (SEM, Apreo 2S, Thermo Scientific). Elemental mapping was performed using energy‐dispersive X‐ray spectroscopy (EDS, QUANTAX, Brucker). The crystal structure of LDHs and GA‐HBC‐L were characterized by X‐ray diffraction with Cu Kα radiation (XRD, X'pert PRO MPD).

### Performance Evaluation—Injectability and Self‐Healing of the Hydrogels

The inverted bottle method was employed to observe the time required for the visual transition of hydrogel flowability, facilitating a quantitative evaluation of the crosslinking rate. Initially, the sol‐gel conversion and injectability of HBC and GA‐HBC hydrogels were assessed at a macroscopic level. The hydrogel solution was prepared in 5 mL single‐channel syringes and stored at 4 °C before being injected into various molds and placed in 37 °C water. The GA‐HBC hydrogel was cut into two halves, and observations were made after reattachment at the fracture surface.

### Mechanical Testing of the Hydrogels

The mechanical properties of HBC and GA‐HBC hydrogels were investigated using a dynamic thermomechanical analyze (DMA 1, METTLER TOLEDO). For tensile tests, rectangular hydrogel specimens with dimensions of ≈20 mm (length) × 4 mm (width) × 0.8 mm (thickness) were used, and a stretch rate of 5 mm min^−1^ was applied. All the tensile tests were conducted at room temperature (25 °C) and humidity of ≈60%. Subsequently, cylindrical samples with a diameter of 8 mm and height of 10 mm were prepared for compression tests at a constant compression rate of 5 mm min^−1^ and room temperature (25 °C). Shear adhesion strength tests were then conducted on the two groups of hydrogels. Pig skin pieces with dimensions of ≈15 mm (length) × 15 mm (width) × 2 mm (thickness) were attached to the hydrogels and placed in an environment at 37 °C for 5 min. Finally, the two pieces of pig skin were slowly separated at a rate of 5 mm min^−1^ to determine the adhesion strength.

### Rheological Testing of the Hydrogels

To streamline the preparation process, it was omitted loading ICA into the hydrogels due to its extremely low concentration and minimal impact on their rheological behavior. The rheological characteristics of each group of hydrogel samples were evaluated using a rheometer (DRH‐2, TA Discovery). First, the repeatability of the self‐healing process was evaluated through cyclic strain experiments with strains of 1% and 100%, with a strain interval of 60 s. Second, to determine the isothermal frequency‐dependent storage modulus (G′) and loss modulus (G″), all hydrogel samples were measured at a strain of 1% and a frequency range of 0.1–100 Hz at a constant temperature of 37 °C. Subsequently, the shear‐thinning properties of the hydrogels were evaluated by testing the shear‐dependent viscosity within a shear rate range of 0.1 to 1000 s^−1^ at 37 °C. Strain scans were performed on cylindrical hydrogel samples with a diameter of 25 mm and a height of 10 mm. Finally, dynamic temperature scanning tests were conducted over a heating rate of 1 °C min^−1^, a frequency of 1 Hz, and a strain of 1%, within the temperature range of 15–45 °C, to detect the storage modulus (G′) and loss modulus (G″). The intersection temperature of G′ and G″ was recorded as the phase transition temperature for each group of hydrogel samples.

### Degradation and Swelling Behavior of the Hydrogels

To investigate the in vitro degradation behavior of hydrogels, the hydrogel samples were initially weighed and recorded as W_0_. Subsequently, the samples were immersed in 5 mL of PBS and rotated at a constant temperature of 37 °C at a rate of 100 rpm. Fresh PBS was replaced daily. At predetermined time points (2, 4, 6, 8, 10, 12, and 14 days), the remaining hydrogel samples were weighed and recorded as W_t_. The degradation rate was calculated using the following formula:

(1) $$\mathrm{Remaining}\;\mathrm{Ratio}=W_{t}/W_{0}\times 100\%$$

Hydrogels were equilibrated in PBS (pH 7.4) for 24 h and then weighed to obtain the wet weight (W_wet_). Subsequently, the hydrogels were lyophilized for an additional 24 h to obtain freeze‐dried hydrogels, and their dry weight (W_dry_) was recorded. The equilibrium water content was determined using the following formula:

(2) $$\mathrm{Water}\;\mathrm{Content}(\%)=(W_{\mathrm{wet}}-W_{\mathrm{dry}})/W_{\mathrm{wet}}\times 100\%$$

The swelling behavior of the hydrogels was evaluated using the equilibrium swelling index. Initially, freeze‐dried hydrogels were weighed (W_dry_) and then immersed in PBS (pH 7.4) at 37 °C on a horizontal shaker set to 100 rpm until swelling equilibrium was reached. Subsequently, the hydrogels were removed from the solution, excess liquid was blotted with filter paper, and the swollen weight (W_swollen_) was recorded. The swelling index of hydrogels incorporated with varying concentrations of LDHs was quantified using the following formula:

(3) $$\mathrm{Swelling}\;\mathrm{Index}=(W_{\mathrm{swollen}}-W_{\mathrm{dry}})/W_{\mathrm{dry}}$$

### ICA and Zn^2+^ Release Behavior of the Hydrogels

Using a UV–vis spectrophotometer, the characteristic absorption peaks of the supernatant before and after ICA loading were analyzed. Meanwhile, ICA was directly incorporated into the GA‐HBC hydrogel (GA‐HBC@ICA) as a control. Subsequently, the hydrogels were immersed in 5 mL of PBS solution (pH 7.4) and subjected to constant agitation at 30 rpm and 37 °C. At specified time intervals, the supernatant was collected and replaced with an equal volume of fresh PBS. The ICA content in the supernatant was calculated and a drug release profile was constructed.

Initially, the release kinetic of Zn^2^⁺ from the GA‐HBC‐LIC hydrogel was assessed. The GA‐HBC‐LIC hydrogel was immersed in a shaker at 37 °C with a speed of 100 rpm. Supernatant samples were collected at specified intervals and replaced with an equivalent volume of fresh PBS. The mean concentration of Zn^2+^ in the collected supernatant was quantified using ICP‐AES. Subsequently, the hydrogel was submerged in 5 mL of DI water and PBS (pH 6.5), respectively, and the supernatant was collected on days 3 and 7 to analyze the cumulative release of Zn^2+^. Finally, GA‐HBC and GA‐HBC‐LIC hydrogels were immersed in 5 mL of DI water. The medium was refreshed daily, and the pH of the supernatant was measured using a pH meter (Sartorius PB‐10).

### Antioxidant Efficiency of the Hydrogels

Add the DPPH solution (0.2 mM, in ethanol) to 100 µL various hydrogel samples. The reaction mixture should be incubated in the dark at 37 °C for 30 min. Subsequently, measure the absorbance of the solution at 517 nm to determine the scavenging rate of DPPH free radicals by the hydrogels. The percentage of DPPH scavenging capacity is calculated using the following formula:

(4) $$\begin{array}{ccc} \mathrm{DPPH}\;\mathrm{Inhibition}(\%) & = & [1-(\mathrm{Absorbance}\;\mathrm{of}\;\mathrm{sample}/\\ & & \mathrm{Absorbance}\;\mathrm{of}\;\mathrm{control})]\times 100\% \end{array}$$

The CAT‐like capacity of the samples was validated using titanium sulfate colorimetry. Specifically, 100 µL of various hydrogel samples were incubated with hydrogen peroxide (H_2_O_2_, 500 µM) in PBS solution at 37 °C for 15 min, followed by centrifugation to collect the supernatant. The subsequent assay procedures were conducted according to the Hydrogen Peroxide Assay Kit. To detect the content of hydroxyl radicals (•OH), the Hydroxyl Radical Assay Kit was employed. First, 100 µL hydrogel samples were reacted with the reagents provided in the kit and then centrifuged to retain the supernatant. Finally, the absorbance of the supernatant was measured at 536 nm using a Microplate Reader, and the •OH scavenging rate was calculated based on these data.

### Cell Culture

Mouse embryonic osteoblast cells (MC3T3) and human gingival epithelial cells (HGE) were cultured in α‐MEM medium, while mouse leukemia cells of monocyte macrophage (Raw264.7) were cultured in DMEM medium. All cell cultures were maintained in their respective media supplemented with 10% FBS and 1% penicillin‐streptomycin. For initiating osteogenic differentiation in MC3T3 cells, the Osteogenic Differentiation Induction (ODI) medium was used. This medium consisted of α‐MEM supplemented with 10% FBS, 1% penicillin‐streptomycin, 10 mM β‐glycerophosphate, 50 µg mL^−1^ L‐ascorbic acid, and 100 nM dexamethasone. The extract medium was prepared by immersing composite hydrogels (1 mL each) in 10 mL of culture medium for 24 h. Prior to use, all hydrogel samples were sterilized under UV and ozone conditions for 4 h.

### Cytocompatibility Assay

HGE, Raw264.7, and MC3T3 cells were co‐cultured with diverse materials for specific durations (1, 2, and 3 days), and the culture medium was substituted with a serum‐free medium containing 10% CCK‐8, and the absorbance at 450 nm was gauged using a microplate reader to determine cell viability. After 24 h co‐culture, Calcein AM (5 µM) and PI (10 µM) were used to determine the live‐dead status of the cells. Images were captured by a Nikon microscope, where live cells were stained green with Calcein AM and dead cells were stained red with PI.

Additionally, a hemolysis assay was performed to evaluate the blood compatibility of the hydrogels. Briefly, erythrocytes were isolated from whole blood through repeated centrifugations and diluted to 2% (v/v) in normal saline.100 µL of hydrogel samples were added to 1 mL the erythrocyte suspension and incubated at 37 °C for 4 h. Then, the tubes were centrifuged at 3000 rpm for 10 min. Photographs of the RBC mixture in the tubes were taken. The OD value at 540 nm of the supernatant was measured, and the hemolysis rate was calculated using the following formula:

(5) $$\begin{array}{ccc} \mathrm{Hemolysis}\;\mathrm{Rate}(\%) & = & [({\mathrm{OD}}_{\mathrm{sample}}{-\mathrm{OD}}_{\mathrm{negative}\;\mathrm{control}})/\\ & & (OD_{\mathrm{positive}\;\mathrm{control}}-OD_{\mathrm{negative}\;\mathrm{control}})]\times 100\% \end{array}$$

where deionized water and saline were utilized as the positive and negative controls, respectively.

### Antibacterial Assay

*Staphylococcus aureus* (*S.aureus*, ATCC29213) was cultured in LB medium at 37 °C under normoxic conditions. The periodontal pathogen *Porphyromonas gingivalis* (*P. gingivalis*, ATCC 33 277) was cultured in BHI medium at 37 °C under anaerobic environment. The OD_600_ _nm_ value of the bacterial suspensions was measured to monitor bacterial growth. Once the OD_600_ _nm_ value reached 0.5, indicating a concentration of ≈1 × 10^8^ CFU mL^−1^.

After co‐incubating the bacterial suspension (1 × 10^8^ CFU mL^−1^, 1 mL) with 1 mL of the hydrogel sample for 1, 2, and 3 days, the OD_600_ _nm_ value of the supernatant was measured to assess cell viability. Following a 24 h co‐culture of the bacterial suspension (1 × 10^8^ CFU mL^−1^, 1 mL) with 1 mL of the hydrogel, 10 µL of the bacterial solution was withdrawn and diluted 100‐fold using PBS buffer. The diluted solution was then spread onto blood agar plates and incubated at 37 °C for 12 h for colony counting. To evaluate the integrity of bacterial membranes, bacteria were co‐cultured with various hydrogels for 24 h. After co‐culture, the hydrogels were removed and washed with PBS, then fixed with 2.5% glutaraldehyde. Subsequently, the samples were dehydrated with a series of ethanol concentrations and vacuum dried. Bacterial morphology in each group of samples was examined and captured using SEM.

For biofilm cultivation, 1 mL of *S. aureus* and *P. gingivalis* (1 × 10^8^ CFU mL^−1^) was incubated in a 24‐well plate upon hydrogels at 37 °C for 48 h. Subsequently, the culture medium was removed, and the unattached bacteria were gently washed away with three rinses of PBS, leaving the biofilm visible at the bottom of the well. Crystalline violet staining and quantitative analysis were performed on the various biofilm samples. Additionally, bacterial viability staining was conducted using a Live & Dead Bacterial Staining Kit. Fluorescence images were captured using a laser scanning confocal microscope (Nikon AxR, Japan).

### In Vitro Study—Osteogenic Induction

Hydrogels were injected into a 96‐well plate, followed by seeding 1 × 10^5^ MC3T3 cells on the hydrogel surface. The CCK8 assay was used to assess the influence of different samples on the proliferation of MC3T3 cells after 1, 2, and 3 days. Additionally, the cells were stained with Calcein AM and PI, and images were captured using a Nikon microscope. 1 × 10^5^ MC3T3 cells were seeded in a 24‐well plate and cultured in osteogenic medium containing hydrogel extracts for 14 days. Osteogenic differentiation was evaluated via alkaline phosphatase (ALP) staining and alizarin red staining (ARS), with results analyzed using a Nikon microscope. ALP expression was quantified using the BCIP/NBT reagent kit. Mineralization was assessed by adding acetic acid and measuring the absorbance at 405 nm for ARS analysis.

### qRT‐PCR Analysis

Gene expression of inflammation‐related markers (*TNF‐α*, *IL‐1β*, *IL‐10*, and *IL‐4*) and osteogenesis‐related markers (*ALP*, *RUNX2*, *Collagen I*, and *Sp7*) was determined by real‐time quantitative polymerase chain reaction (qRT‐PCR). 1 × 10^5^ Raw264.7 cells were co‐cultured with various hydrogels for 48 h. After co‐culture, the cells were collected using Trizol reagent, and total RNA was extracted. 1 × 10^5^ MC3T3 cells were cultured in the supernatant of the aforementioned Raw264.7 cells along with ODI medium for 14 days. The MC3T3 cells were then collected using Trizol reagent, and total RNA was extracted. cDNA was synthesized using a Reverse Transcription Kit. qPCR was performed using the SYBR Green Detection System kit (n = 3). The primers were listed in Table S4 (Supporting Information).

Gene expression of immune regulation‐related markers (*ZEB1*, *IL‐6*, and *IL‐10*) and osteogenesis‐related markers (*ALP* and *RUNX2*) was assessed by qRT‐PCR. 1 × 10^5^ Raw264.7 cells were co‐cultured with the GA‐HBC‐LIC hydrogel, Mocetinostat (a ZEB1 inhibitor), or GA‐HBC‐LIC hydrogel combined with Mocetinostat in the presence of LPS. Simple LPS induction served as the periodontitis group, while the blank control group received no treatment. Following the same procedure as described above, 1 × 10^5^ MC3T3 cells were cultured with the supernatant of the aforementioned Raw264.7 cells and ODI medium. The subsequent processing methods were consistent with those mentioned previously. The primers were listed in Table S4 (Supporting Information).

### Intracellular ROS Scavenging and Zn^2+^ Staining

DCFH‐DA (10 µM) in FBS‐free medium was used for intracellular reactive oxygen species (ROS) examination. After incubation, the solution was removed and cells were washed twice with PBS. Fluorescent images were captured using a Nikon microscope, followed by quantitative analysis using ImageJ software. For intracellular Zn^2+^ testing, TSQ (1 µg mL^−1^) in PBS was employed.

### Cytoskeleton Staining

1 × 10^5^ Raw264.7 cells were co‐cultured with various hydrogels at 37 °C for 24 h. Simultaneously, 100 ng ml^−1^ LPS and 20 ng ml^−1^ IL‐4 were used to induce M1 and M2 polarization of the cells, respectively. Subsequently, the cells were fixed with 4% paraformaldehyde and permeabilized with 0.5% Triton X‐100. Finally, the cytoskeleton and nucleus of the macrophages were stained with FITC‐phalloidin and DAPI, and the morphological changes were observed using a Nikon microscope.

### Western Blot Assay

In addition, Western blot analysis was performed to evaluate the protein expression of ALP, RUNX2, Collagen I, Sp7, and GAPDH. Specifically, 1 × 10^5^ MC3T3 cells were grown in a conditioned medium for 14 days. Then, the cells were collected and lysed using WB/IP lysis buffer at 4 °C for 10 min. Next, 40 µg of protein from each sample was loaded onto a 10% SDS‐PAGE gel and electrophoresed at 80 V for 1.5 h. Proteins were transferred onto a PVDF membrane. After blocking with 5% milk for 2 h, the membrane was incubated with primary antibodies at 4 °C overnight. The primary antibodies used were as follows: ALP (Mouse, 1:750), RUNX2 (Mouse, 1:750), Collagen I (Mouse, 1:750), Sp7 (Mouse, 1:750), and GAPDH (Mouse, 1:45000). Subsequently, the membrane was incubated with secondary antibodies (Mouse, 1:3000) at room temperature for 2 h. Protein bands were visualized using an ECL Chemiluminescent Substrate Kit, followed by quantitative analysis using ImageJ software.

Additionally, Western blot analysis was conducted to evaluate the protein expression of ZEB1, BMP‐2, VEGF, and β‐actin. 1 × 10^5^ Raw264.7 cells were co‐cultured with various samples in the presence of LPS. Simple LPS induction served as the periodontitis group, while the blank control group received no treatment. 1 × 10^5^ MC3T3 cells were grown in a conditioned medium for 14 days. The subsequent processing methods were consistent with those mentioned previously. Primary antibodies used were as follows: ZEB1 (Mouse, 1:750), BMP‐2 (Mouse, 1:750), VEGF (Mouse, 1:750), and β‐actin (Mouse, 1:45000).

### Transcriptome Sequencing

The gene expression profiles of macrophages cultured on different hydrogels were analyzed using RNA sequencing (RNA‐seq). Specifically, 1 × 10^5^ Raw264.7 cells were co‐cultured on PBS (as a control), HBC, GA‐HBC, GA‐HBC‐IC, GA‐HBC‐L, and GA‐HBC‐LIC hydrogels for 24 h, with 100 ng mL^−1^ LPS added to construct an inflammatory microenvironment. Cells were lysed in Trizol reagent, and mRNA was extracted. The isolated RNA samples were subjected to RNA‐seq, and the data were analyzed Hangzhou Lianchuan Biotechnology Co., Ltd. (https://www.omicstudio.cn/index). Three biological replicate samples were used for RNA‐seq analysis in each group.

### In Vivo *Study*

All animal experiments were conducted in accordance with the guidelines for the use and care of laboratory animals approved by the Ethics Committee of the Biological Resource Centre at the Agency for Science, Technology and Research, Zhejiang University, under approval code ZJU20241149. Male Balb/c mice, 8 weeks old, were purchased from Shanghai SLAC Laboratory Animal Co., Ltd. All animals were treated humanely throughout the experiments.

### Degradation Evaluation

Hydrogel samples were subcutaneously injected into the backs of mice, and the weight of the implanted hydrogel was determined as the difference in the syringe weight before and after injection (designated as W_0_). At each time point (0, 2, and 4 weeks), macroscopic photographs of the implantation site were taken. To assess degradation performance, the hydrogel samples were retrieved from the subcutaneous sites of the mice after 4 weeks, photographed, and weighed (designated as W_t_). The degradation rate was calculated using the following formula:

(6) $$\mathrm{Remaining}\;\mathrm{Ratio}=W_{t}/W_{0}\times 100\%$$

### Periodontitis Model and Hydrogel Injection

8‐week‐old male Balb/c mice were reared in a specific pathogen‐free (SPF) environment with a 12 h light/dark cycle and provided with sterile food and water ad libitum. The mice were randomly divided into seven groups (n = 10). After one week of adaptive feeding, they were anaesthetized with pentobarbital sodium (40 mg kg^−1^), and 5‐0 surgical sutures were ligated and fixed around the upper second molar of each mouse. Except for the blank control group, the remaining groups received continuous injections of *P. gingivalis* (10 µL, 10^5^ CFU mL^−1^)for one week.^[^ ^59^ ^]^ Two weeks later, the ligatures were removed to confirm the successful establishment of the periodontitis model. Different hydrogel samples (50 µL) were injected into the submucosal periosteal tissue at the buccal and palatal midpoints of the upper second molar using a micro syringe, while the blank group was injected with 50 µL of PBS. For in vivo immune regulation studies, the mice were randomly divided into five groups and processed according to the aforementioned methods. After one week of treatment with the hydrogel in each group, Mocetinostat was administered intravenously (i.v.) three times per week for three consecutive weeks.

### Micro‐CT Analysis

Micro‐computed tomography (Micro‐CT) scans were performed on the upper jaw samples (SCANCO µCT 100, Scanco Medical, Switzerland; source voltage: 70 kVp, power: 200 µA, exposure time: 500 ms, and voxel size: 30 microns) to evaluate alveolar bone (AB) resorption in mice subjected to different treatments. The raw scan data were reconstructed, and 3D bone formation analysis was performed using the Scan Evaluation software. Key parameters assessed included bone volume/total volume (BV/TV), local bone mineral density (BMD), trabecular number (Tb. N), trabecular thickness (Tb. Th), and trabecular spacing (Tb. Sp). Additionally, buccal and palatal periodontal bone heights were measured as the distance from the cementoenamel junction (CEJ) to the alveolar bone crest (ABC) in the interdental region.

### Histology and Immunohistochemistry

After the Micro‐CT scan, the upper jaw samples were fixed in 4% paraformaldehyde for 48 h, then decalcified, dehydrated, embedded, and sectioned using standard histological procedures. Hematoxylin and eosin (H&E) and Masson trichrome staining were performed to evaluate histological changes and collagen synthesis in the periodontal tissue at 4 weeks post‐treatment. All sections were captured using a bright‐field light microscope (Eclipse Ci‐L, Nikon, Japan). On the 4th week after modeling, immunofluorescence staining was employed to detect the expressions of iNOS and CD206, allowing observation of macrophage infiltration and polarization at the defect site. Immunohistochemical staining was utilized to assess the expressions of OPN, BMP‐2, and VEGF in the periodontal bone defect region 4 weeks after hydrogel injection, to evaluate the effect on tissue regeneration. Quantitative analyses of each staining result were conducted using ImageJ software.

### qRT‐PCR Analysis

Complete maxillae containing maxillary teeth were collected from mice, and the expression levels of *TNF‐α*, *IL‐10*, *TGF‐β*, *BMP‐2*, *VEGF*, *OCN*, and *OPN* were determined by qRT‐PCR (n = 3). Total RNA was extracted from maxillary tissues using Trizol reagent and reverse‐transcribed into cDNA using a Reverse Transcription Kit. qPCR was performed using the SYBR Green Detection System kit. The primer sequences were listed in Table S4 (Supporting Information).

### Western Blot Assay

Western blot analysis was performed on the maxillary tissues of mice to evaluate the expression levels of ZEB1, BMP‐2, VEGF, and β‐actin. Complete maxillae containing maxillary teeth were collected, crushed, and subjected to tissue lysis. The supernatant was retained after centrifugation. Subsequent processing methods were consistent with those previously described. The primary antibodies used were as follows: ZEB1 (mouse, 1:750), BMP‐2 (mouse, 1:750), VEGF (mouse, 1:750), β‐actin (mouse, 1:45000), CD86 (mouse, 1:750), CD206 (mouse, 1:750), ALP (mouse, 1:750), RUNX2 (mouse, 1:750), and GAPDH (mouse, 1:45000).

### Biosafety Assessment

Upon completion of the experiment, major organs (heart, liver, spleen, lung, and kidney) were collected for H&E staining and microscopic examination using a Nikon microscope. The in vivo biosafety of the hydrogel was evaluated. Routine blood tests were conducted to measure white blood cell (WBC) count, red blood cell (RBC) count, hemoglobin (HGB) concentration, mean corpuscular volume (MCV), platelet (PLT) count, and mean corpuscular hemoglobin concentration (MCHC). Blood biochemical tests were conducted to assess liver function (alanine aminotransferase (ALT), alkaline phosphatase (ALP), aspartate aminotransferase (AST)) and kidney function (blood urea nitrogen (BUN), creatinine (CREA), uric acid (UA)).

### Statistical Analysis

Bioinformatic analyses, including volcano plot, heat map, Gene Ontology (GO) enrichment analysis, Kyoto Encyclopedia of Genes and Genomes (KEGG) enrichment analysis, Gene Set Enrichment Analysis (GSEA) enrichment analysis, and Principal Component Analysis (PCA) analysis were performed using OmicStudio tools (https://www.omicstudio.cn/tool) with default parameters (fold change ≥ 1.5; *p < 0.05*). All data are presented as the mean ± standard deviation and were analyzed using Origin 2024 (64‐bit). Comparisons and significance analyses among multiple groups were conducted using Student's t‐test. Statistical significance was defined as ***p < 0.001, **p < 0.01, and *p < *0.05*.

## Conflict of Interest

The authors declare no conflict of interest.

## Supporting information



Supporting Information

Supplemental Video 1

Supplemental Video 2

## Data Availability

The data that support the findings of this study are available from the corresponding author upon reasonable request.
