# Supplementary material for: Customized Hydrogel System for the Spatiotemporal Sequential Treatment of Periodontitis Propelled by ZEB1
Source: Adv Sci (Weinh). 2025 Apr 4;12(26):2503338. doi: 10.1002/advs.202503338 (PMC12245124; doi:10.1002/advs.202503338)
Supplement: Supplementary file 1 — Supporting Information [file ADVS-12-2503338-s002.docx]

**Supporting Information**

**Customized Hydrogel System for the Spatiotemporal Sequential Treatment of Periodontitis Propelled by ZEB1**

Jiafei Chen^1#^, Xiaoxu Guan^1#^, Lina Chen^3#^, Bingzhu Zheng^6^, Feiyu Li^2^, Chao Fang^5^, Yike Fu^2,4^, Xiang Li^2*^, Huiming Wang^1*^, Yi Zhou^1*^

This part includes 4 tables, 2 videos, and 26 figures.

| **Sample** | **Molecular Formula** | **Zn/Al** |
| --- | --- | --- |
| Zn_3_Al_1_-LDHs | Zn_0.2706_Al_0.1529_ | 1.76:1 |

Table S1: Chemical composition of ZnAl-LDHs based on ICP analysis results.

| The Composition of Injectable Thermoresponsive Hydrogel | | | |
| --- | --- | --- | --- |
| Sample | Hydroxybutyl Chitosan | Gallic Acid | ZnAl-LDHs |
| HBC | 5% (wt/vol) | × | × |
| GA-HBC | √ | 7.5% (wt/vol) | × |
| GA-HBC-L0.5 | √ | √ | 0.005% (wt/vol) |
| GA-HBC-L1 | √ | √ | 0.01% (wt/vol) |
| GA-HBC-L1.5 | √ | √ | 0.015% (wt/vol) |
| GA-HBC-L2 | √ | √ | 0.02% (wt/vol) |

**Table S2:** The composition of various LDHs-incorporated hydrogel samples (HBC, GA-HBC, GA-HBC-L0.5, GA-HBC-L1, GA-HBC-L1.5 and GA-HBC-L2).

| The Composition of Injectable Thermoresponsive Hydrogel  for In Vitro and In Vivo Studies | | | | |
| --- | --- | --- | --- | --- |
| Sample | Hydroxybutyl Chitosan | Gallic Acid | ZnAl-LDHs | Icariin |
| HBC | √ | × | × | × |
| GA-HBC | √ | √ | × | × |
| GA-HBC-IC | √ | √ | × | √ |
| GA-HBC-L | √ | √ | √ | × |
| GA-HBC-LIC | √ | √ | √ | √ |

**Table S3:** The composition of injectable thermoresponsive hydrogel samples (HBC, GA-HBC, GA-HBC-IC, GA-HBC-L, and GA-HBC-LIC) was evaluated for both in vitro and in vivo studies.

| **Gene Name** | **Forward Primer (5’-3’)** | **Reverse Primer (5’-3’)** |
| --- | --- | --- |
| *ALP* | TCCTGACCAAAAACCTCAAAGG | TGCTTCATGCAGAGCCTGC |
| *RUNX2* | GGTACTTCGTCAGCATCCTATCAG | GCTTCCGTCAGCGTCAACAC |
| *Collagen Ⅰ* | TCCCTACTCAGCCGTCTGTG | CCTCGCTTCCGTACTCGAAC |
| *SP7* | CAAAGAAGCCATACGCTGACCT | AGGAAATGAGTGAGGGAAGGGT |
| *BMP-2* | GGACCCGCTGTCTTCTAGTG | TTCCTCGATGGCTTCTTCGT |
| *TGF-β* | GCAACAATTCCTGGCGTTACC | CAGTGAGCGCTGAATCGAAAG |
| *VEGF* | TCCGTAGTAGCCGTGGTCT | CCTCTCCTCTTCCTTCTCTTCC |
| *OPN* | AGCAAGAAACTCTTCCAAGCAA | GTGAGATTCGTCAGATTCATCCG |
| *OCN* | CTGACCTCACAGATGCCAAGC | TGGTCTGATAGCTCGTCACAAG |
| *TNF-α* | CTCAGCGAGGACAGCAAGG | AGGGACAGAACCTGCCTGG |
| *IL-6* | GGACTGATGCTGGTGACAAC | GGAGTGGTATCCTCTGTGAAGT |
| *IL-1β* | TTCAAGGGGACATTAGGCAG | TGTGCTGGTGCTTCATTCAT |
| *IL-4* | GGTCTCAACCCCCAGCTAGT | GCCGATGATCTCTCTAAGTGAT |
| *IL-10* | CCTGGATCTGTATCACCGAAGC | CTCCGACCACTCTGCCTTGTTA |
| *ZEB1* | TTATCCTGAGGCGCCCGAGGA | TACGGGCAGGTGAGCAACTGG |
| *GADPH* | CCTCGTCCCGTAGACAAAATG | TGAGGTCAATGAAGGGGTCGT |

**Table S4:** List of primers used for this study.

**Video S1:** Video of the injection of GA-HBC hydrogel into water at 37 °C.

**Video S2:** Video of the GA-HBC hydrogel on the skin surface being washed away by running water.


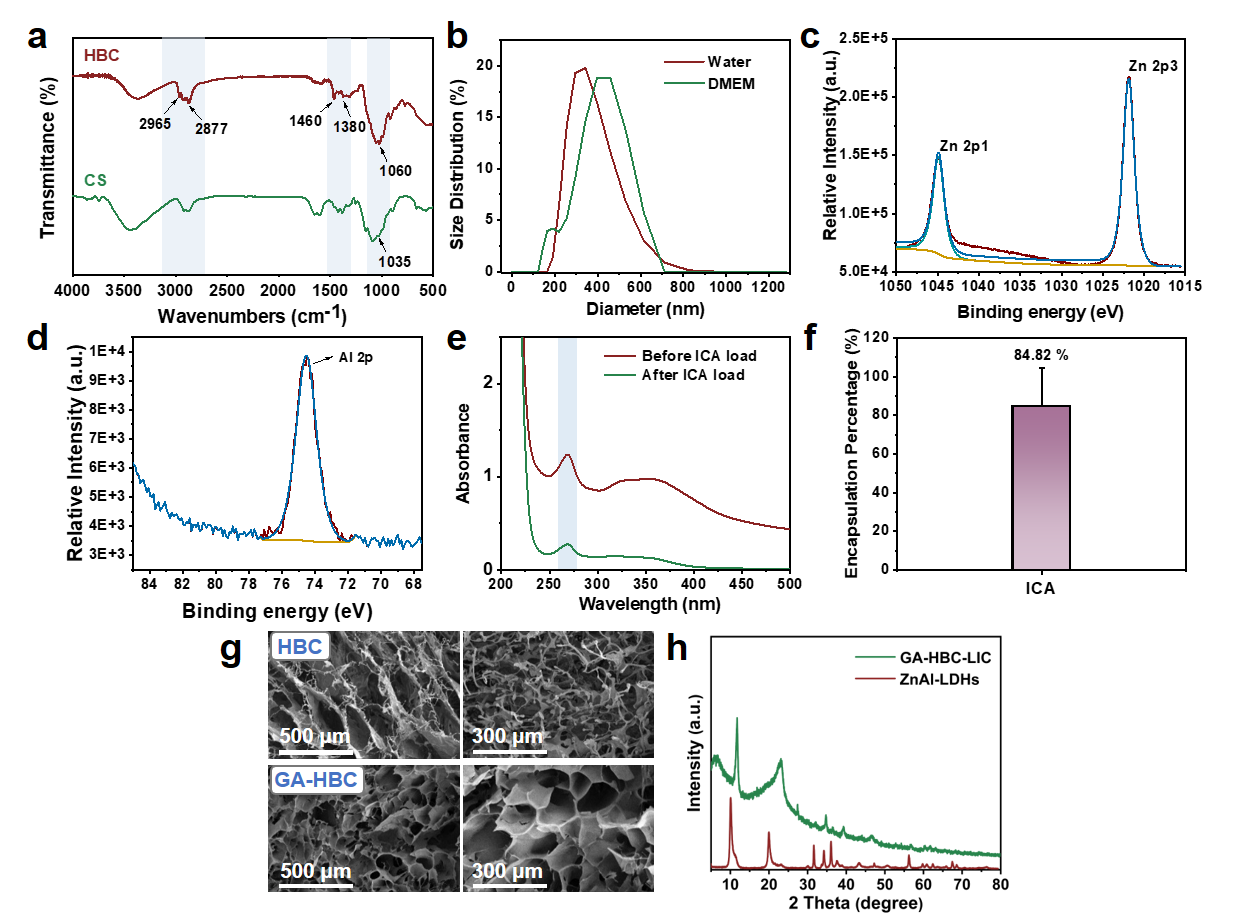


**Figure S1** a) FT-IR spectra of CS and HBC. b) Size distribution of ZnAl-LDHs in physiological solvents (water and DMEM). High-resolution XPS spectra of Zn 2p (c) and Al 2p (d). e) UV-Vis spectra before and after ICA loading. f) Loading capacity of ICA into ZnAl-LDHs. g) SEM micrographs of HBC and GA-HBC hydrogels. h) XRD spectra of ZnAl-LDHs and the GA-HBC-LIC hydrogel.


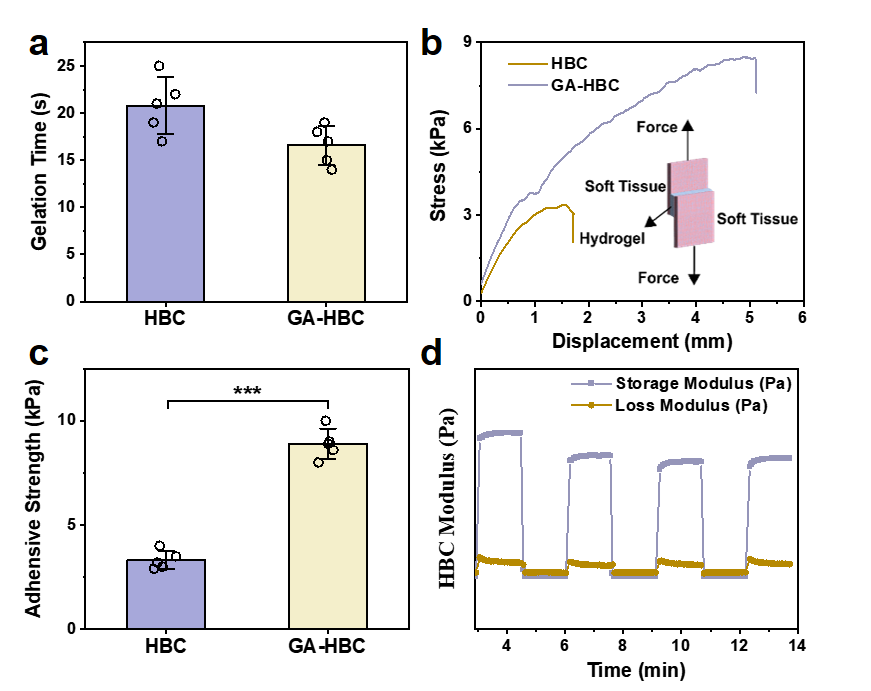


**Figure S2** a) Gelation time of HBC and GA-HBC hydrogels. Force-displacement curves (b) and adhesion strength (c) of HBC and GA-HBC hydrogels. d) Step-strain sweep of HBC hydrogel. ****p < 0.001*.


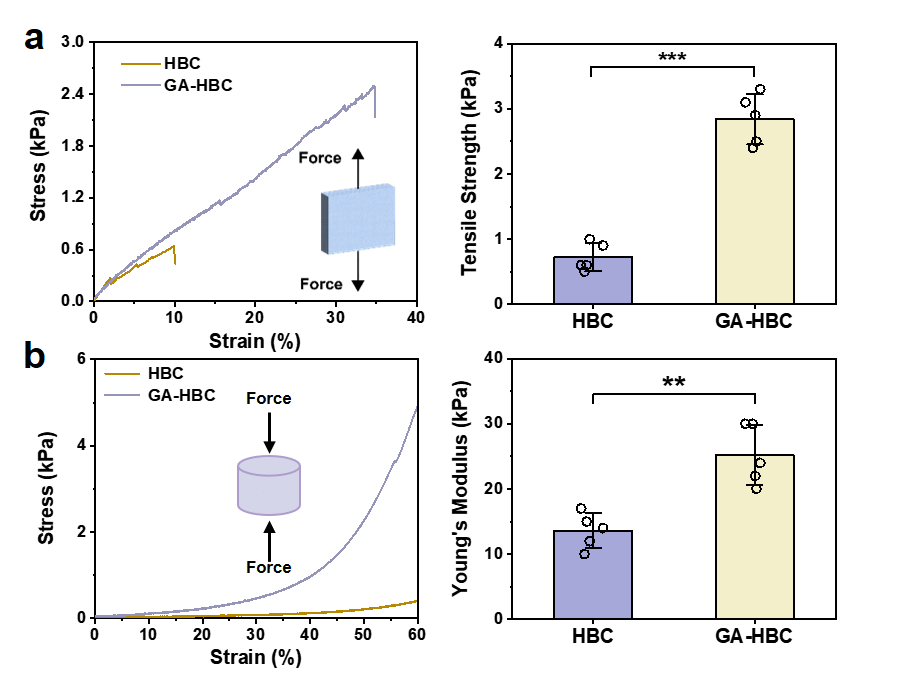


**Figure S3** a) Stress-strain curves and tensile strength of HBC and GA-HBC hydrogels. b) Stress-strain curves and Young's modulus of HBC and GA-HBC hydrogels. ***p < 0.01, ***p < 0.001*.


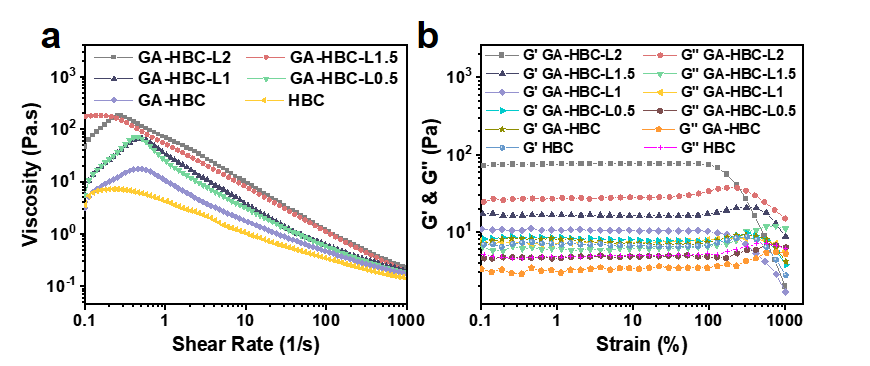


**Figure S4** a) Viscosity-shear rate of various hydrogels at 37 °C. b) Strain-scan curves in the rheological characterization of various hydrogels.


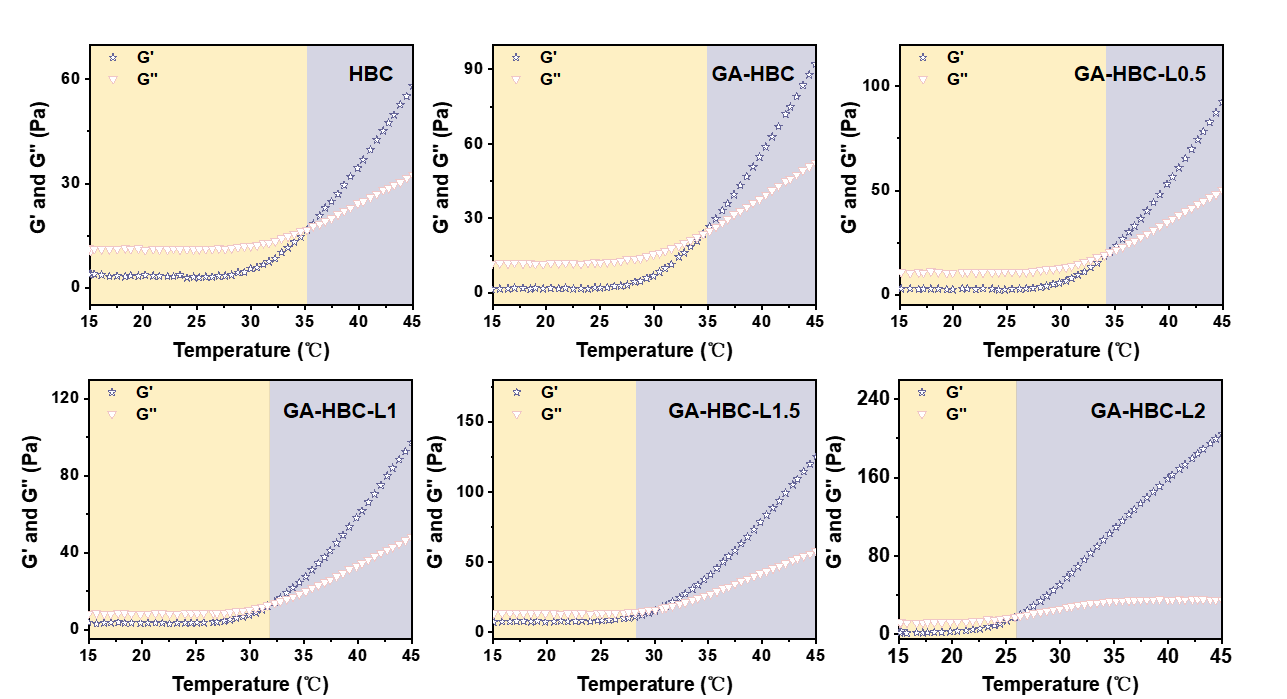


**Figure S5** Typical temperature-dependent functions of G’ and G” for different samples.


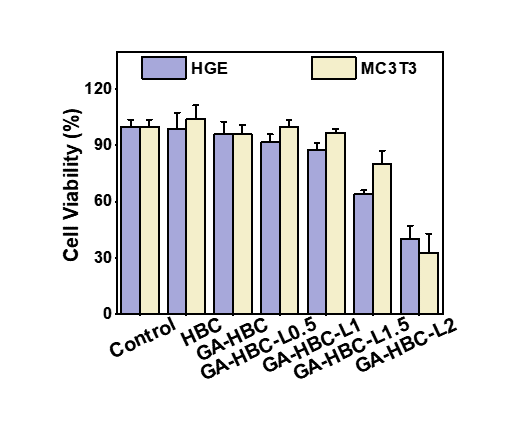


**Figure S6** Cell viability of HGE cells and MC3T3 cells after treatment with different hydrogels.


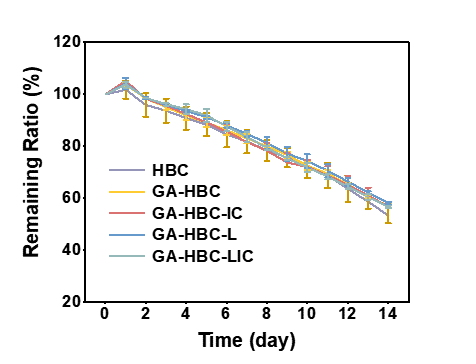


**Figure S7** Degradation behavior of various hydrogels.


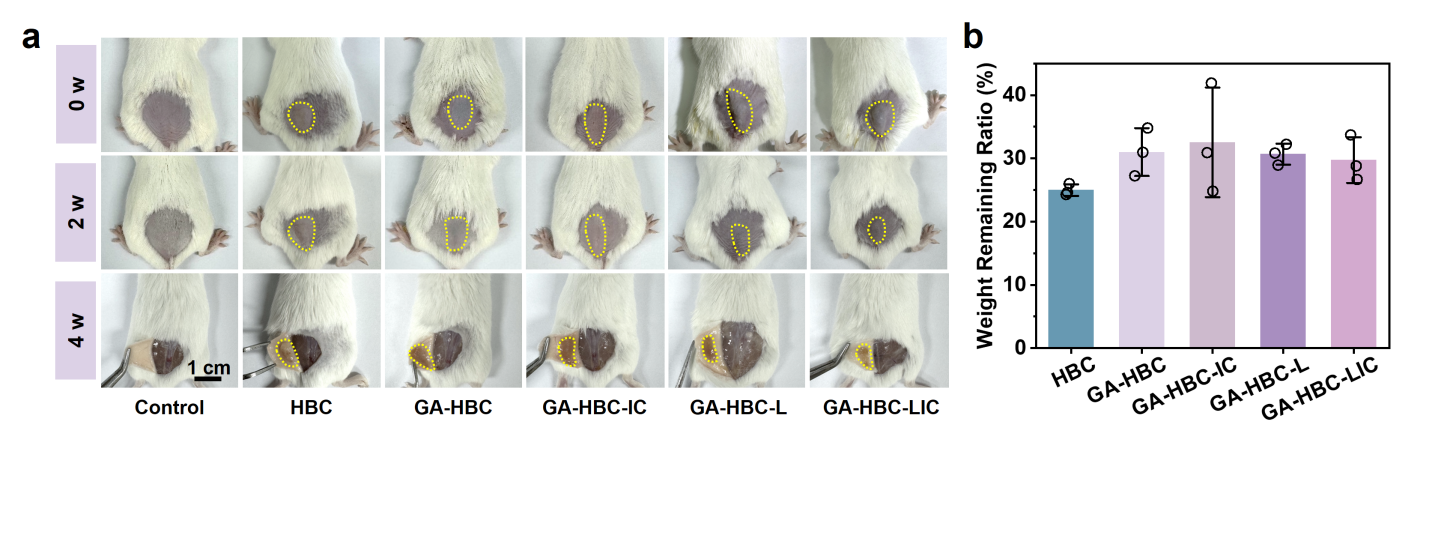


**Figure S8** a) Images of subcutaneous degradation in mice at 0, 2, and 4 weeks post-treatment. b) Weight remaining ratio of the hydrogels in the subcutaneous tissue of mice after 4 weeks of treatment.


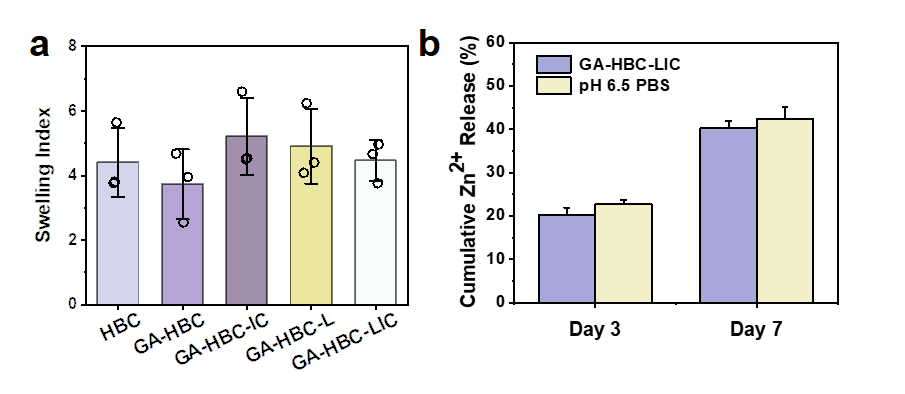


**Figure S9** a) Swelling index of different samples. b) Cumulative amount of Zn^2+^ released from the GA-HBC-LIC hydrogel and PBS solution at the 3th and 7th days.


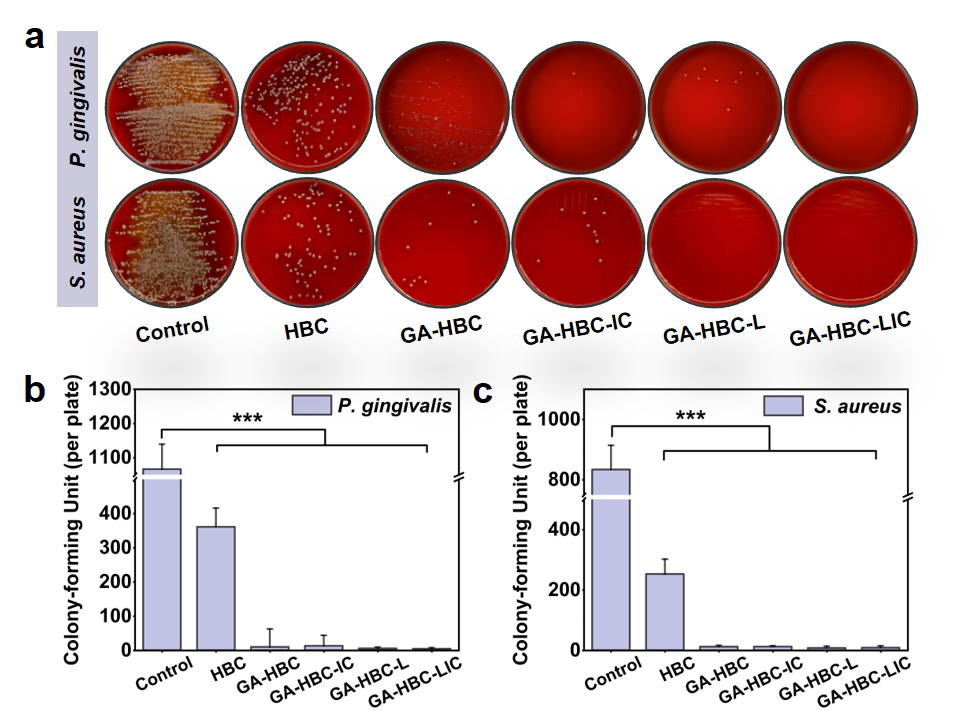


**Figure S10** a) Representative agar plate images of *P. gingivalis* and *S. aureus* treated with various hydrogels. b) Quantitative analysis of colony-forming units (CFUs) for *P. gingivalis* and *S. aureus* subjected to various hydrogel treatments. ****p < 0.001*.


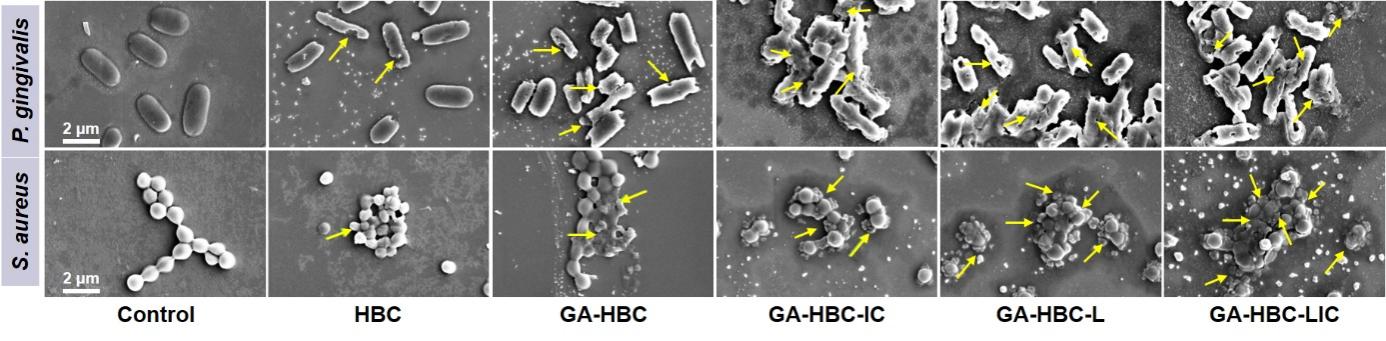


**Figure S11** SEM images of *P. gingivalis* and *S. aureus* after treatment with various hydrogels (Yellow arrows indicate the irregular morphology of bacterial cells).


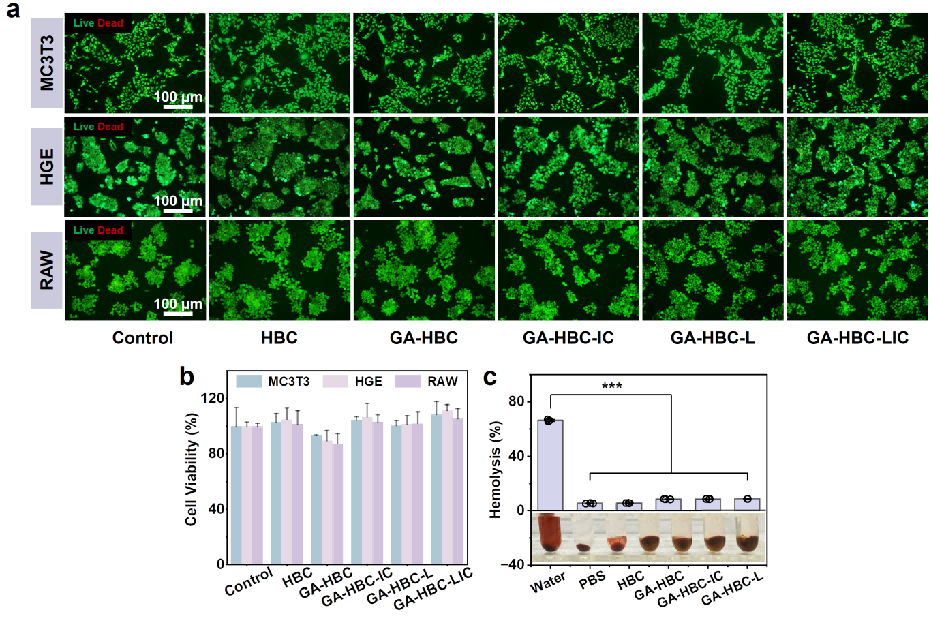


**Figure S12** a) Live/Dead staining of MC3T3, HGE, and Raw264.7 cells after incubation with various hydrogels. b) Cell viability of MC3T3, HGE, and Raw264.7 cells treated with extracts from different hydrogels. c) Hemolytic percentage of the hydrogels (Illustration shows a photograph of the hemolysis test). ****p < 0.001*.


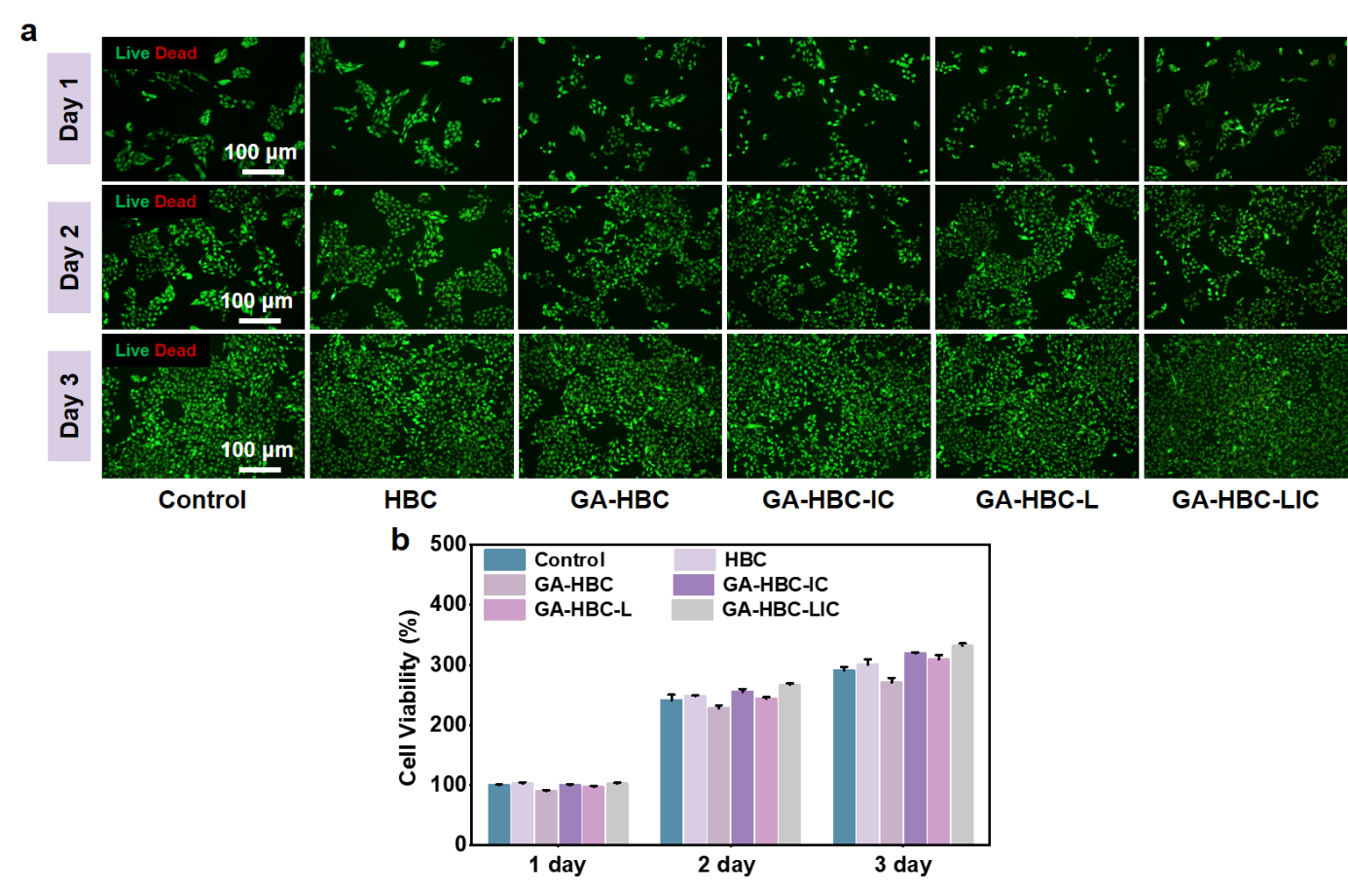


**Figure S13** a) Live/Dead staining of MC3T3 cells after incubation with various hydrogels for 1, 2, and 3 days. b) Cell viability of MC3T3 cells after incubation with various hydrogels for 1, 2, and 3 days.


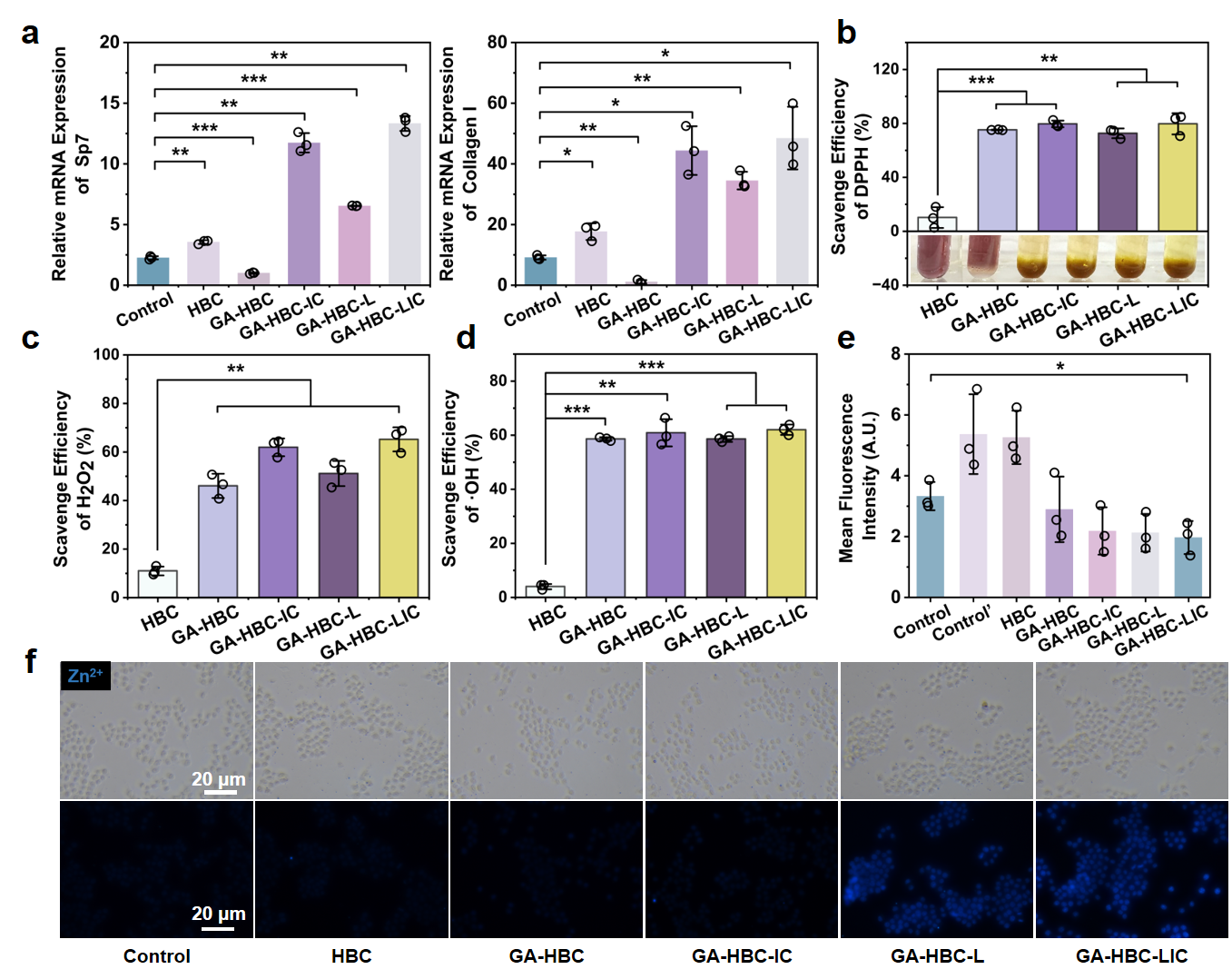


**Figure S14** a) Relative mRNA expression of osteogenesis-related genes at 14 days. b) DPPH-scavenging capability of various hydrogels (Illustrative photograph of the scavenging ability experiment). H_2_O_2_ (c) and •OH (d) scavenging capability of various hydrogels. e) Statistical analysis of relative cell fluorescence intensity based on DCFH staining. f) TSQ probe staining for Zn^2+^ detection. **p < 0.05, **p < 0.01, ***p < 0.001*.


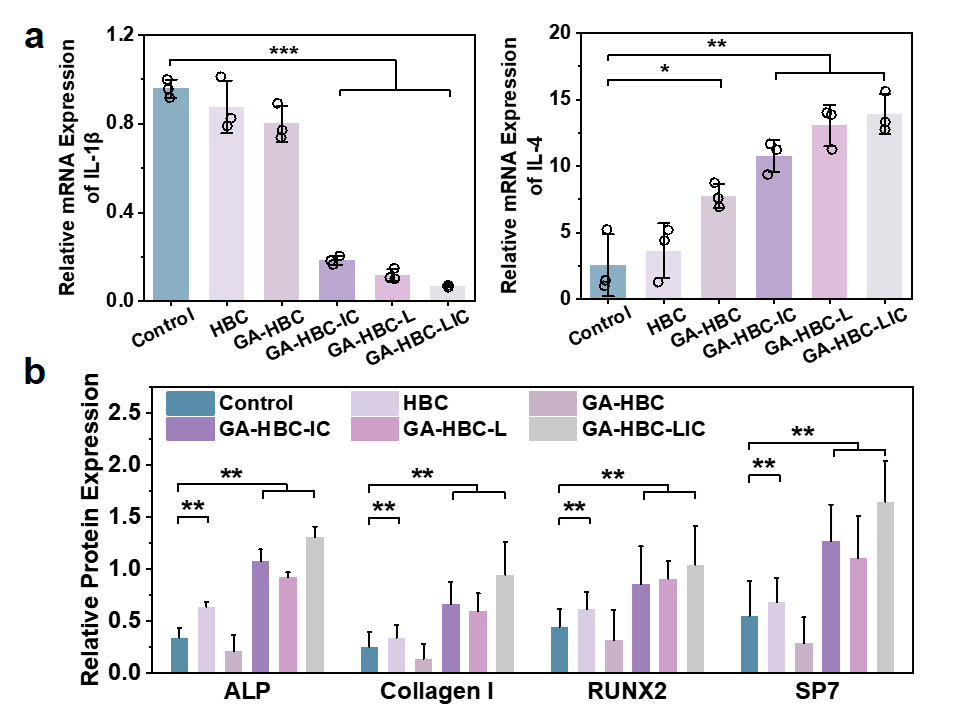


**Figure S15** a) Relative mRNA expression of inflammation-related genes. b) Quantitative analysis of ALP, SP7, RUNX2, and Collagen I protein expression. **p < 0.05, **p < 0.01, ***p < 0.001*.


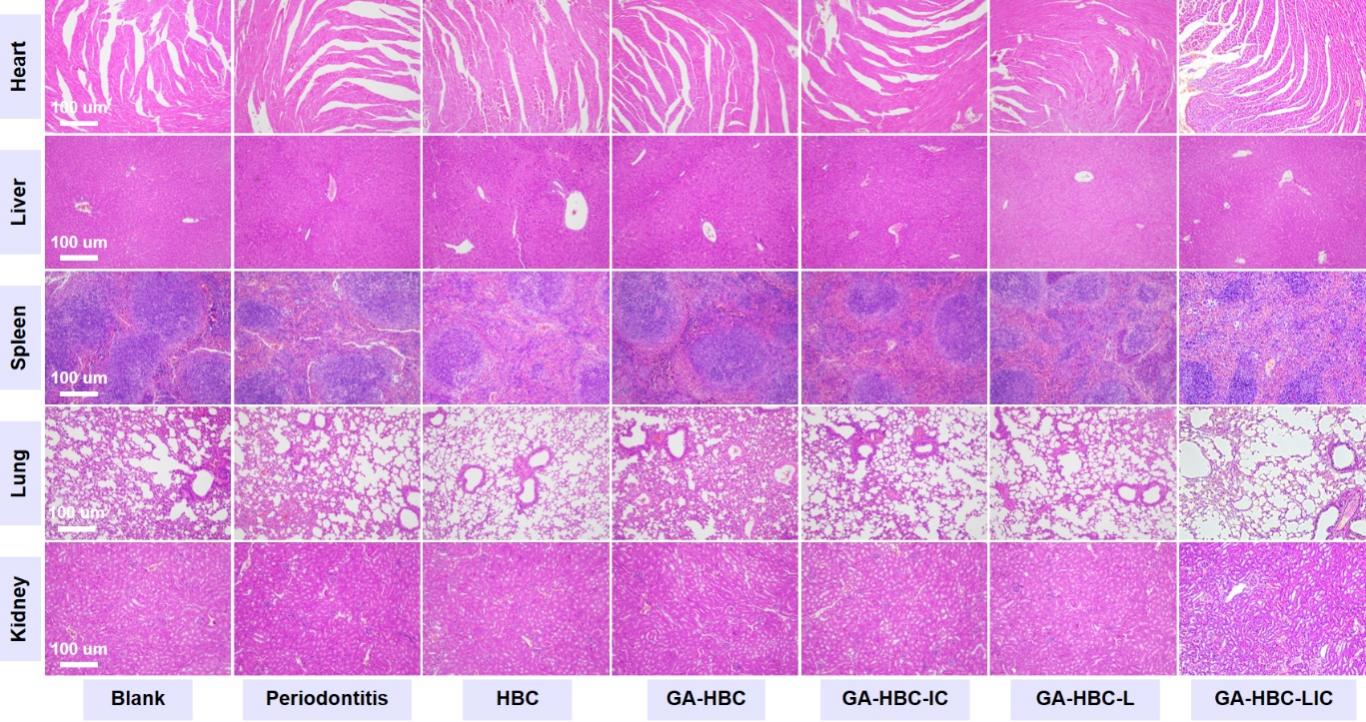


**Figure S16** H&E staining of major organ sections (heart, liver, spleen, lung, and kidney) from each group.


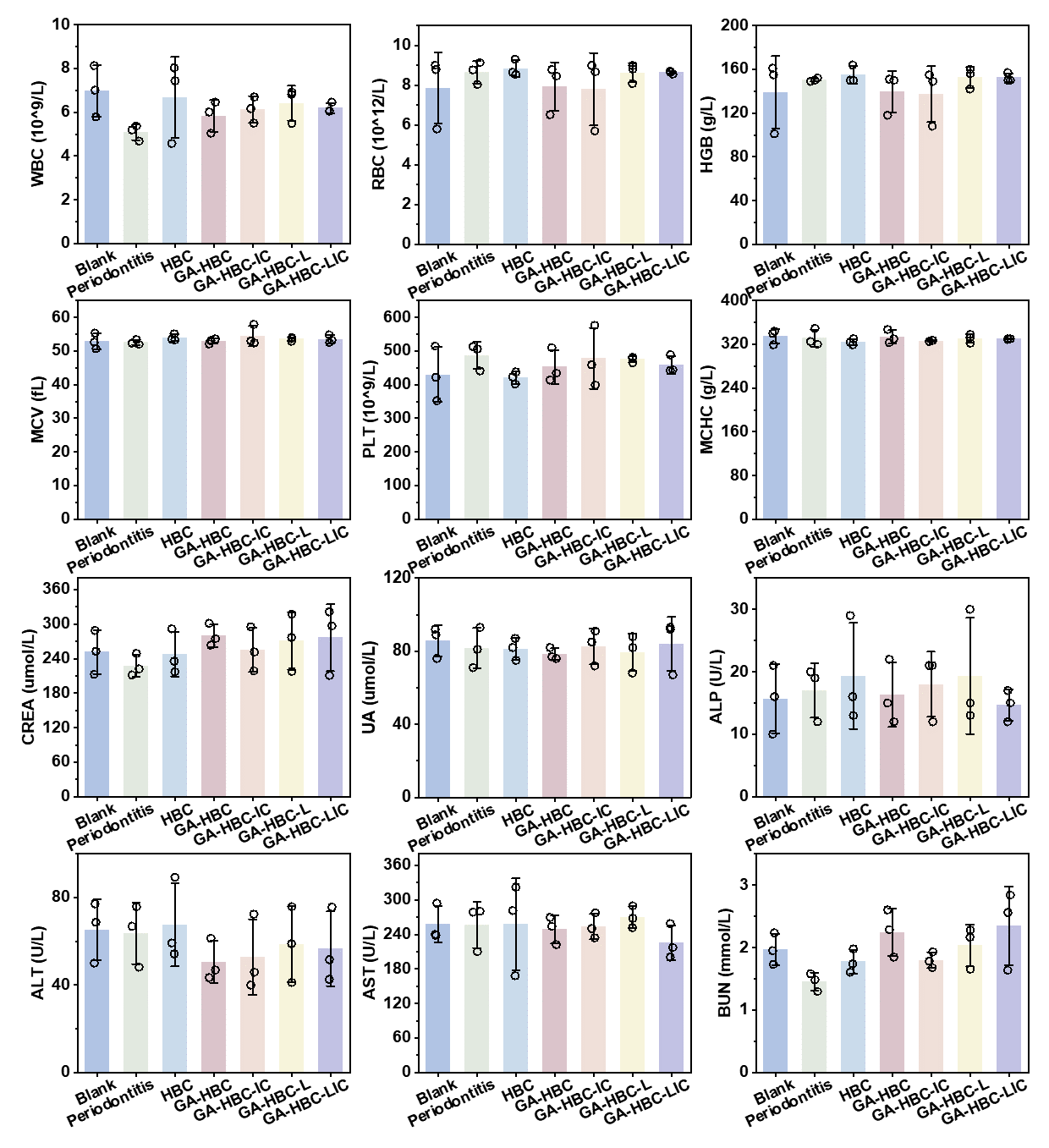


**Figure S17** White blood cell (WBC), red blood cell (RBC), hemoglobin (HGB), mean corpuscular volume (MCV), platelets (PLT), mean corpuscular hemoglobin concentration (MCHC), creatinine (CREA), uric acid (UA), alkaline phosphatase (ALP), alanine aminotransferase (ALT), aspartate aminotransferase (AST), and blood urea nitrogen (BUN) levels in mice after 4 weeks of different treatments.


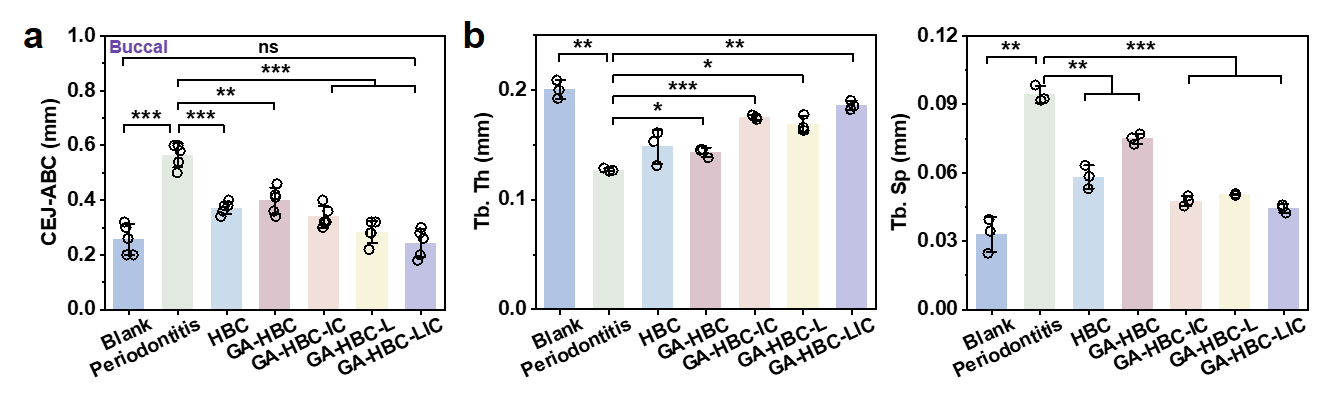


**Figure S18** a) Quantitative analysis of buccal bone resorption and bone formation (CEJ-ABC) in each group. b) Trabecular thickness (Tb. Th) and trabecular spacing (Tb. Sp) were assessed using Micro-CT quantitative analysis. **p < 0.05, **p < 0.01, ***p < 0.001*.


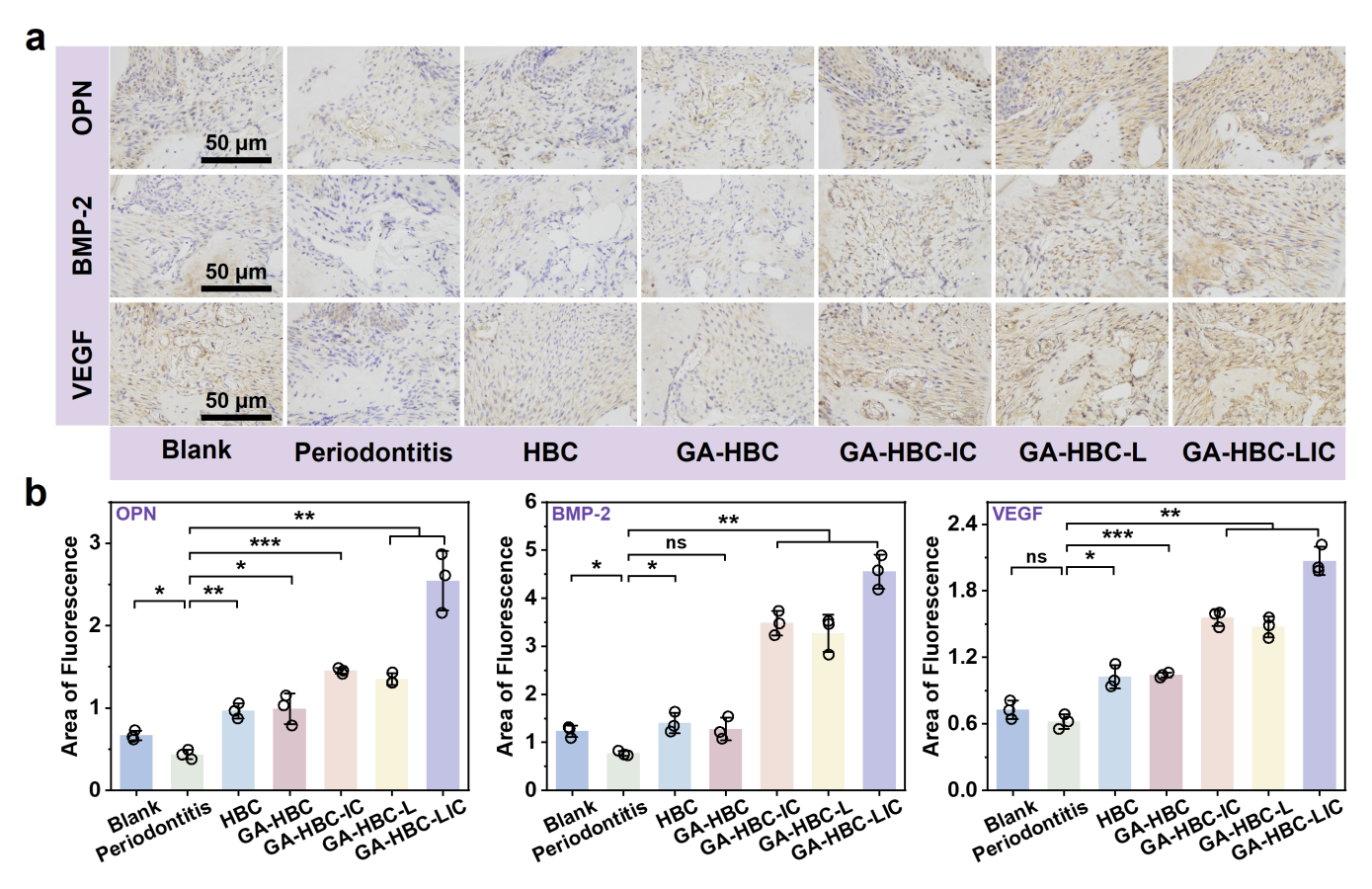


**Figure S19** a) Immunohistochemical staining of OPN, BMP-2, and VEGF in each group. b) Quantitative analysis of the mean integral optical density (IOD) for OPN, BMP-2, and VEGF. **p < 0.05, **p < 0.01, ***p < 0.001*.


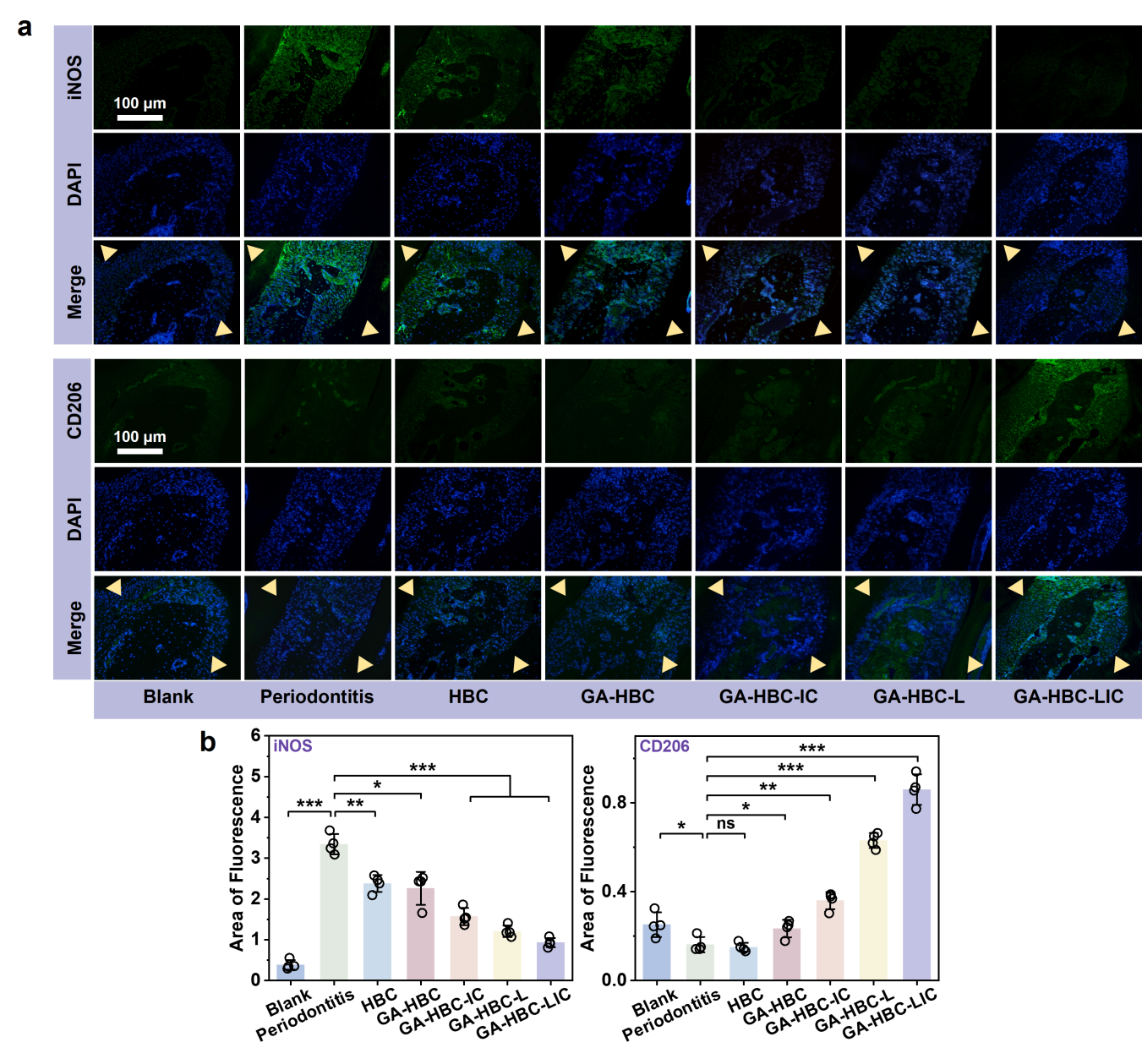


**Figure S20** a) Immunofluorescence staining of iNOS and CD206 in each group. b) Quantitative image analysis of the fluorescence intensity of iNOS and CD206. **p < 0.05, **p < 0.01, ***p < 0.001*.


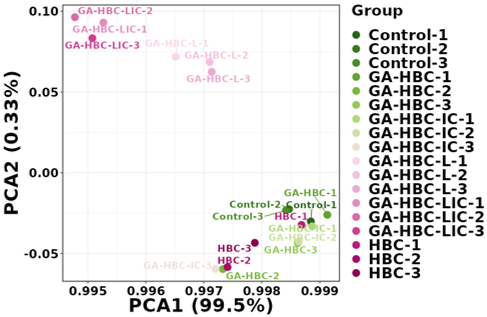


**Figure S21** Principal coordinate analysis of Raw264.7 cells after different treatments.


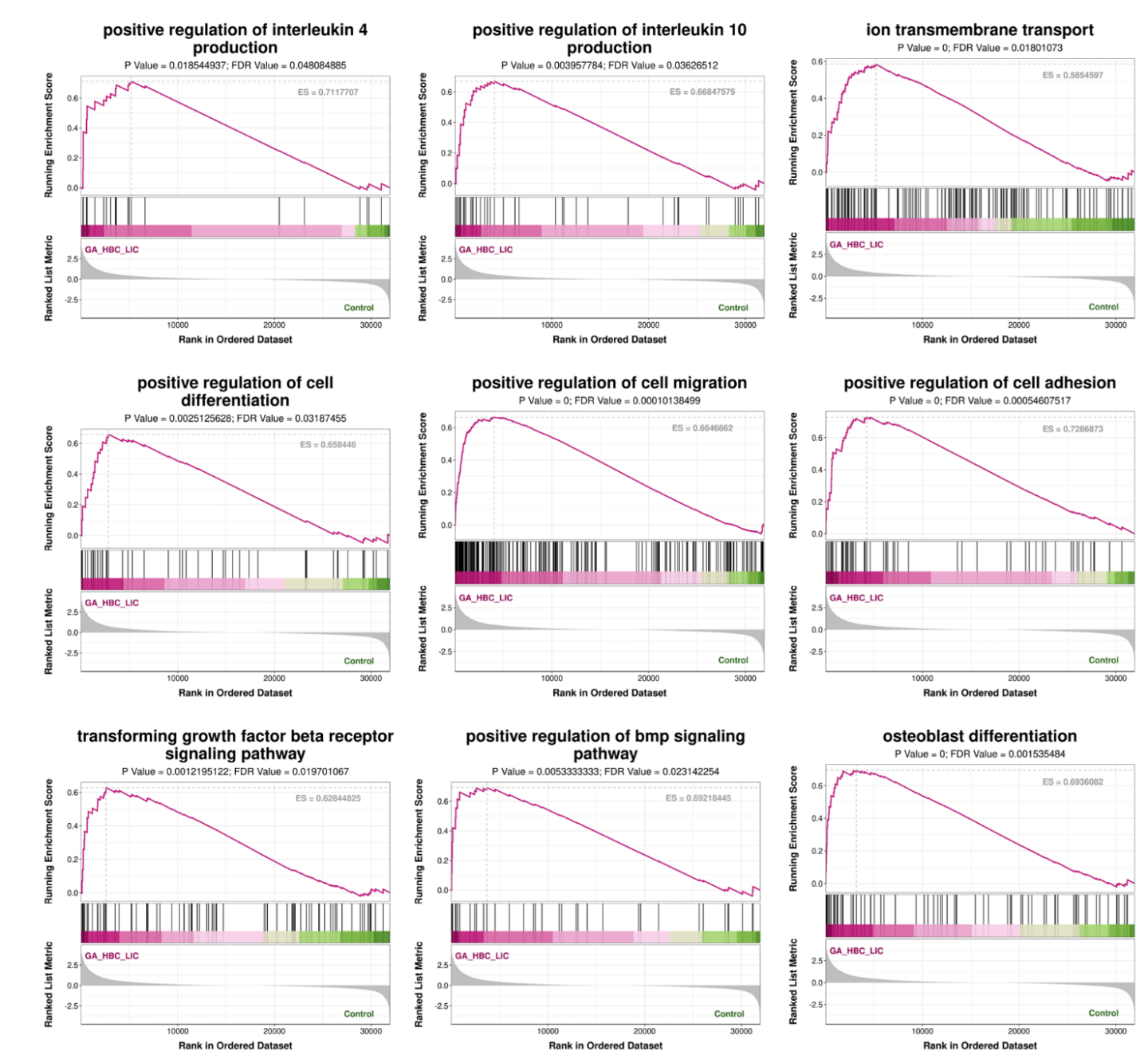


**Figure S22** GSEA of pathways following RNA-seq.


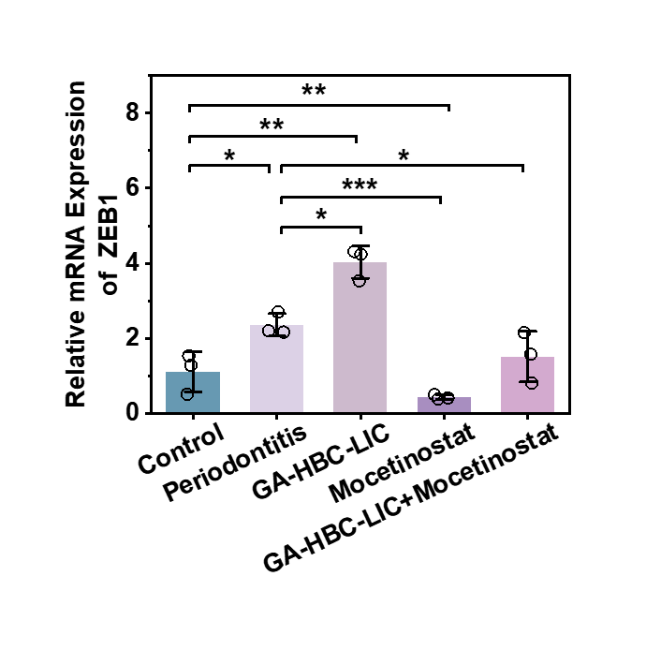


**Figure S23** Relative mRNA expression levels of ZEB1 in the Raw264.7 cells following different treatments. **p < 0.05, **p < 0.01, ***p < 0.001*.


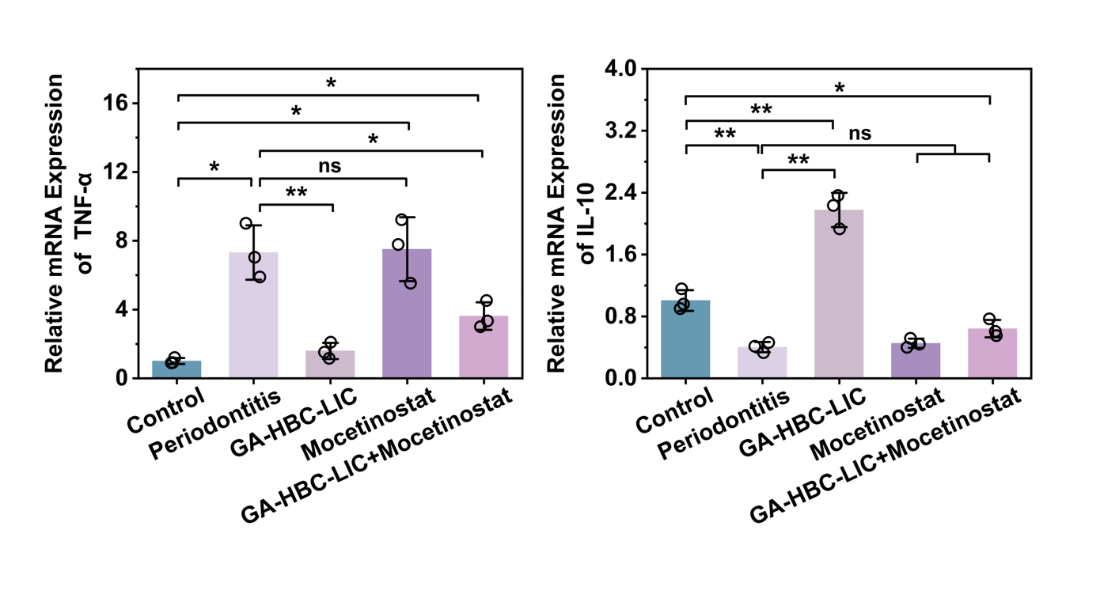


**Figure S24** Relative mRNA expression levels of inflammation-related genes in maxillary tissues following different treatments. **p < 0.05, **p < 0.01*.


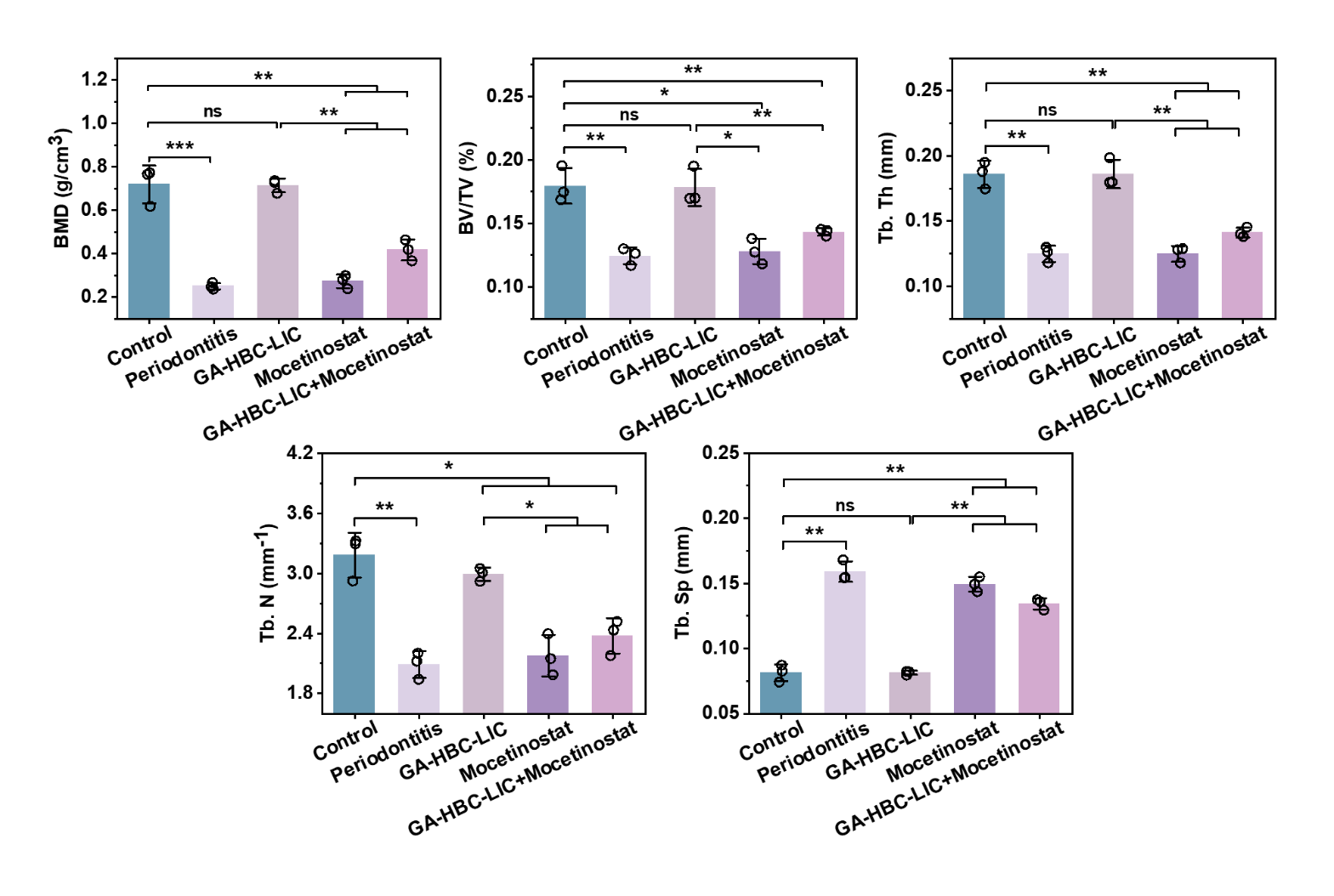


**Figure S25** Bone mineral density (BMD), bone volume/total volume (BV/TV), trabecular thickness (Tb. Th), trabecular number (Tb. N), and trabecular spacing (Tb. Sp) were quantified using Micro-CT analysis. **p < 0.05, **p < 0.01, ***p < 0.001*.


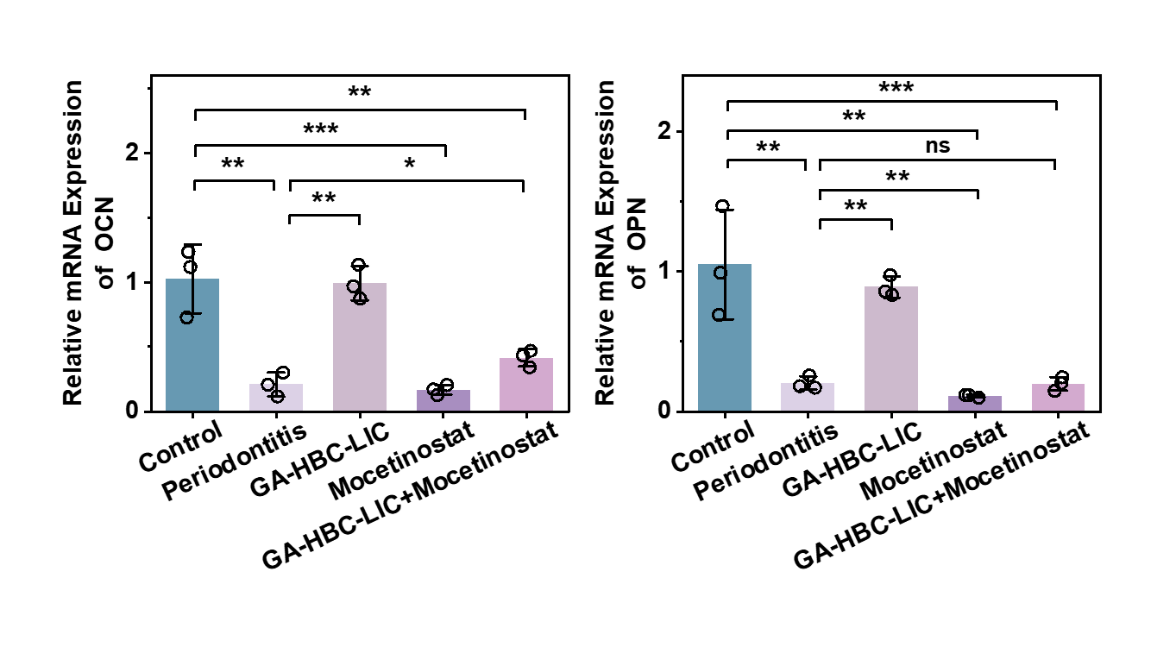


**Figure S26** Relative mRNA expression levels of osteogenesis-related genes in maxillary tissues following different treatments. **p < 0.05, **p < 0.01, ***p < 0.001*.
